# Transcriptome Mining Provides Insights into Cell Wall Metabolism and Fiber Lignification in *Agave tequilana* Weber

**DOI:** 10.3390/plants11111496

**Published:** 2022-06-02

**Authors:** Luis F. Maceda-López, Elsa B. Góngora-Castillo, Enrique Ibarra-Laclette, Dalia C. Morán-Velázquez, Amaranta Girón Ramírez, Matthieu Bourdon, José L. Villalpando-Aguilar, Gabriela Toomer, John Z. Tang, Parastoo Azadi, Jorge M. Santamaría, Itzel López-Rosas, Mercedes G. López, June Simpson, Fulgencio Alatorre-Cobos

**Affiliations:** 1 Colegio de Postgraduados, Campus Campeche, Carretera Haltunchén-Edzná km 17.5, Sihochac, Campeche 24450, Mexico; maceda.luis@outlook.com (L.F.M.-L.); carolina.moran21@hotmail.com (D.C.M.-V.); jose.va@china.tecnm.mx (J.L.V.-A.); 2 CONACYT-Centro de Investigación Científica de Yucatán, Unidad de Biotecnología, Calle 43 No. 130 × 32 y 34, Chuburná de Hidalgo, Mérida 97205, Mexico; elsa.gongora@cicy.mx; 3 Red de Estudios Moleculares Avanzados, Instituto de Ecología A. C. Carretera Antigua a Coatepec 351, El Haya, Xalapa 91070, Mexico; enrique.ibarra@inecol.mx; 4 Centro de Investigación Científica de Yucatán, Unidad de Biotecnología, Calle 43 No. 130 × 32 y 34, Chuburná de Hidalgo, Mérida 97205, Mexico; amaranta.giron@cicy.mx (A.G.R.); jorgesm@cicy.mx (J.M.S.); 5 Sainsbury Laboratory, University of Cambridge, Cambridge CB2 1LR, UK; matthieu.bourdon@slcu.cam.ac.uk; 6 Division of Microbiology and Molecular Biology, IIT Research Institute, Chicago, IL 60616, USA; gtoomer@iitri.org; 7 Complex Carbohydrate Research Center, University of Georgia, Athens, GA 30602, USA; johnzhoutang@gmail.com (J.Z.T.); azadi@ccrc.uga.edu (P.A.); 8 CONACYT-Colegio de Postgraduados Campus Campeche, Carretera Haltunchén-Edzná km 17.5, Sihochac, Campeche 24450, Mexico; itzel.rosas@colpos.mx; 9 Departmento de Biotecnología y Bioquímica, Centro de Investigación y Estudios Avanzados del IPN, Irapuato 36824, Mexico; mercedes.lopez@cinvestav.mx; 10 Departmento de Ingeniería Genetica, Centro de Investigación y Estudios Avanzados del IPN, Irapuato 36824, Mexico; june.simpson@cinvestav.mx

**Keywords:** agave, cell walls, lignocellulose, CAD protein, CESA protein, sclerenchyma

## Abstract

Resilience of growing in arid and semiarid regions and a high capacity of accumulating sugar-rich biomass with low lignin percentages have placed Agave species as an emerging bioenergy crop. Although transcriptome sequencing of fiber-producing agave species has been explored, molecular bases that control wall cell biogenesis and metabolism in agave species are still poorly understood. Here, through RNAseq data mining, we reconstructed the cellulose biosynthesis pathway and the phenylpropanoid route producing lignin monomers in *A. tequilana*, and evaluated their expression patterns in silico and experimentally. Most of the orthologs retrieved showed differential expression levels when they were analyzed in different tissues with contrasting cellulose and lignin accumulation. Phylogenetic and structural motif analyses of putative CESA and CAD proteins allowed to identify those potentially involved with secondary cell wall formation. RT-qPCR assays revealed enhanced expression levels of AtqCAD5 and AtqCESA7 in parenchyma cells associated with extraxylary fibers, suggesting a mechanism of formation of sclerenchyma fibers in Agave similar to that reported for xylem cells in model eudicots. Overall, our results provide a framework for understanding molecular bases underlying cell wall biogenesis in Agave species studying mechanisms involving in leaf fiber development in monocots.

## 1. Introduction

The cell wall is the boundary between the plant cell and the environment. Its composition affects cell growth, expansion, proliferation, adhesion, and responses to external stimuli [1]. Because the cell wall skeleton is primarily based on fermentable polysaccharides, cell walls are now highly valorized as a sugar source for bioethanol production and other bio-products obtained by biorefinery [2,3,4]. Cellulose, the most abundant polymer on earth, is the major structural polysaccharide of plant primary and secondary cell walls (PCW and SCW, respectively). This compound consists de linear chains of several hundred to many thousands of β (1, 4) linked D-glucose units, which can be defragmented and used for saccharification. Cellulose is up 40–50% and close to 90% in SCW in specialized cells as mature fibers, respectively [5]. However, here, cellulose is found tightly impregnated by lignin, a complex polyphenolic biopolymer that increases the stiffness and strength of SCW but complicates access to cellulose [6]. Understanding lignin and cellulose biosynthesis pathways during SCW formation is critical to dealing with an increasing demand of low-lignin biomass more suitable for producing of second-generation bioethanol from plant biomass.

In Agave species used for spirits production, only pines are used for fermentation, while leaves that account for 40% of total wet weight are considered waste. Agave species are also emerging as biofuel crops because of their drought resilience, adaptation to poor soil, high biomass yield, and notably high cellulose/lignin ratio [7,8,9,10]. Recently, biofuel potential has been evaluated in *A. americana* and *A. deserti*, two species adapted to marginal soils. The results showed biomass with low K-lignin content and enriched with soluble and structural sugars [11], which could be helpful for the bioethanol industry.

Reference genomes have not been reported so far for any Agaves species. However, high-throughput RNA-sequencing has revealed transcriptomic landscapes for several Agave species during responses to environmental stimuli and development. These catalogs of expressed genes include transcriptional profiles of *A. tequilana* Weber, *A. deserti* Engelm, *A. victoriae-reginae* T. Moore, *A. striata* Zucc., *A. americana* L., *A. salmiana* Otto, *A. sisalana* Perr. ex. Engelm, *A. fourcroydes* Lem., and the hybrid H11648 ((*A. amaniensis* Trel and Nowell x *A. angustifolia* Haw) x *A. amaniensis*) [9,12,13,14,15,16,17,18,19]. Because of the economic importance tequila production represents, analysis of RNA-seq data mainly has focused on *A. tequilana* to uncover and characterize sets of genes involved in the fructan biosynthesis and transition to flowering [12,15,16]. Moreover, two Agave species also traditionally used in Mexico since the Precolombin ages, *A. americana* and *A. salmiana*, have been used as models to provide new insights into the molecular mechanisms underlying the CAM metabolism and regeneration in plants, respectively [13,14].

In contrast to eudicots, biosynthetic genes and regulatory network hubs that control the metabolism and deposition of cell wall components have been poorly characterized in monocots. Using comparative genomics and expression profiling analysis, large sets of genes putatively involved in cell wall biogenesis, especially for the components cellulose and lignin, have been reported for maize and rice, but the functional significance of most of those is missing still [20,21,22,23,24,25]. Recently, encouraged by a world economy that is moving back to natural or bio-based fibers, leaf fibers from Agave species have drawn the attention again of industry and science. Transcriptomes of *A. sisalana*, *A. fourcroydes*, and the hybrid H11648, widely cultivated in Brazil, Africa, and China for fiber production, have been explored to know fiber-related genes subjected to positive selection during Agave domestication, those involved with cellulose biosynthesis, and responses to drought [15,17,18,19]. Here, we carried out mining of publicly available RNA-seq data from *A. tequilana* to predict the closest orthologous for the sets of genes involved with cellulose and lignin biosynthesis pathways. Most of the retrieved orthologous genes showed differential expression levels when analyzed in eight transcriptomes from different tissues of *A. tequilana*. Gene expression patterns found in enriched lignocellulosic tissues are in line with the predicted functions for key genes in metabolic pathways of cellulose and lignin. Phylogenetic and structural motif analysis for putative CESA and CAD proteins allowed identifying those potentially involved with SCW formation. RT-qPCR assays revealed enhanced expression levels of AtqCAD5 and AtqCESA7 quantified in parenchymatous cells associated with extraxylary fibers, supporting the accuracy of our orthology analyses and suggesting a mechanism of formation of sclerenchyma fibers in Agave similar to that reported for xylem cells in model eudicots.

## 2. Results

### 2.1. Identification of Orthologous Biosynthetic Genes for Cellulose and Lignin in A. tequilana

Using the orthologous groups obtained by Markov clustering algorithm and a similarity search with Arabidopsis proteins as queries, the closest orthologous genes encoding for all the enzymes involved in the cellulose and lignin pathways were identified in the *A. tequilana* transcriptomes mined (File S1). Gene nomenclature designated for each Agave orthologous was based on gene prefixes previously used (Atq) [12,16,26,27]. For cellulose biosynthesis, all the nine gene families previously annotated and characterized in *A. thaliana*, the best plant model to study cell wall-related genes [28,29], were found expressed in the Agave transcriptomes mined (Table 1). Cellulose Synthase (CESA) and Fructose Kinase (FK) families had the highest number of orthologous genes identified. Ten CESA genes and seven FK genes have been reported in the Arabidopsis genome, while here, we retrieved six and five genes expressed, respectively. Although this study just reflects transcriptomic landscapes, only one Agave orthologous gene was found for *Sucrose Phosphate Synthase* (*SPS*), contrary to those four reported in Arabidopsis. Size, calculated molecular weight, and theoretical isoelectric points were relatively similar within all the gene families analyzed (Table 1), except those for *Fructokinase* (*FK*) and *UDP-Glucose Pyrophosphorylase* (*UGP*) genes. FK1 is 191 aa smaller compared to FK4, while UGP1 has 380 aa fewer with respect to UGP3 (Table 1). For these genes, incomplete open reading frames (ORF) cannot be ruled out. Subcellular localization prediction for most enzymes was consistent with previously reported for carbohydrate metabolism: cytosolic, plastids, or associated to the membrane or cell wall (Table 1) [29,30,31,32]. For example, as expected for plant CESA proteins, all Agave CESA isoforms found in this study were predicted to be targeted to the plasma membrane (Table 1).

Similar to cellulose-related genes, orthologous Agave genes were found for all eleven genes families encoding for enzymes that participate in the biosynthesis of lignin subunits (Table 2). The gene family with more members expressed in the transcriptomes mined was *Cinnamyl Alcohol Dehydrogenase* (*CAD*). Nine *CAD* genes have been functionally characterized in Arabidopsis. Here, seven orthologous *CAD* genes were retrieved in the leaves’ assembled transcriptomes. For the rest of the lignin biosynthetic genes, gene family size was quite similar to that reported in Arabidopsis, except for *Shikimate Hydroxycinnamoyl Transferase* (*HCT*), *Cinnamoyl CoA Reductase* (*CRR*), and *Caffeic Acid 3-O-Methyltransferase* (*COMT*). Interestingly, in *A. tequilana*, more isoforms expressed were found for *HCT*, *CCR*, and *COMT*, respecting those reported in Arabidopsis (Table 2). Predicted protein size and physic-chemical properties were similar among the homologous genes involved with monolignol biosynthesis (Table 2).

### 2.2. Agave Cellulose and Lignin-Related Predicted Genes Are Differentially Expressed in Different Agave Tissues

**Figure 1:**
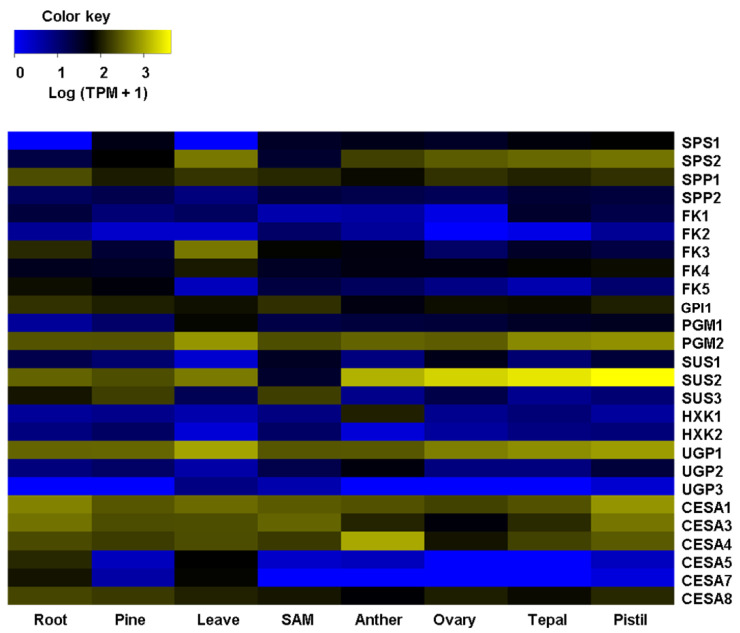
Heatmap depicting expression profiles of orthologous genes involved with cellulose biosynthesis across several tissues/organs of *A. tequilana*. Genes enlisted on the left side are plant biosynthetic genes for cellulose according to the literature. *Sucrose PhosphateSsynthase* (*SPS*), *Sucrose Phosphate Phosphatase* (*SPP*), *Fructokinase* (*FK*), *Phosphoglucomutase* (*PGM*), *Sucrose Synthase* (*SUS*), *Hexokinase* (*HXK*), *UDP-Glucose Ppyrophosphorylase* (*UGP*), and *Cellulose Synthase* (*CESA*). At the bottom, the last row indicates the RNA-sequencing samples previously reported [16]. SAM: Shoot apical meristem. Hierarchical clustering was conducted on the log (TPM+1). In the color key, yellow denotes high expression, black refers to medium, and blue denotes low expression.

Analysis of mRNA abundance for all the biosynthetic orthologous predicted for lignin and cellulose was conducted in Agave transcriptomes previously reported [16]. These RNA-seq data are from several Agave tissues and organs in different developmental stages, and thus, represent a valuable dataset to assess the potential biological roles of the genes identified in this study. For cellulose biosynthesis, all the genes’ isoforms showed differential expression patterns in the eight transcriptomes analyzed that were obtained from tissues highly differentiated as the root, pine, and leaves, or undifferentiated cells of the shoot apical meristem (SAM), for example. Heatmap clustering analysis showed that all the genes encoding enzymes that participate in getting sucrose (Suc) have differential expression levels in the most transcriptomes sequenced (Figure 1, Supplementary Table S3). *Sucrose Phosphate Synthase2* (*AtqSPS2*) and *Sucrose Phosphate Phosphatase1* (*AtqSPP1*) showed medium to high expression in pine and reproductive tissues, while *Fructokinase3* (*AtqFK3*) had similar levels for root and leaves. *Phosphoglucomutase2* (*AtqPGM2*), with a cytosolic localization predicted, showed a broad expression in all the transcriptomes analyzed (Figure 1; Table S1), which is in line with the biological role reported for cytosolic PGM. Plant cytosolic PGM isoforms are involved in Suc catabolism to provide intermediates used in glycolysis and as substrates for synthesizing a variety of cellular constituents [33,34]; thus, it is expected PGMs are ubiquitous in all plant tissues. Among the *three Sucrose Synthase* (*SUS*) isoforms retrieved in our orthology analysis, *AtqSUS2* was the most expressed in all the Agave transcriptomes mined. It was moderately expressed in roots, pines, and leaves and interestingly, highly expressed in reproductive tissues (Figure 1; Table S3). Likewise, *Pgm1*, *UDP-Glucose Pyrophosphorylase1* (*UGP1*), and four *Cellulose Synthase* (*CESA*) isoforms, *AtqCESA1*, *AtqCESA3*, *AtqCESA4*, and *AtqCESA8*, showed a medium expression, fairly uniform, in all the tissues tested (Figure 1; Table S3). Contrary to most of the *CESAs* retrieved, *AtqCESA5* and *AtqCESA7* were found to be expressed only in roots and leaves, which are samples enriched with lignocellulosic cell walls [10,35], suggesting that these two *CESA* orthologous may have a functional role during secondary cell wall biosynthesis in Agave plants.

**Figure 2:**
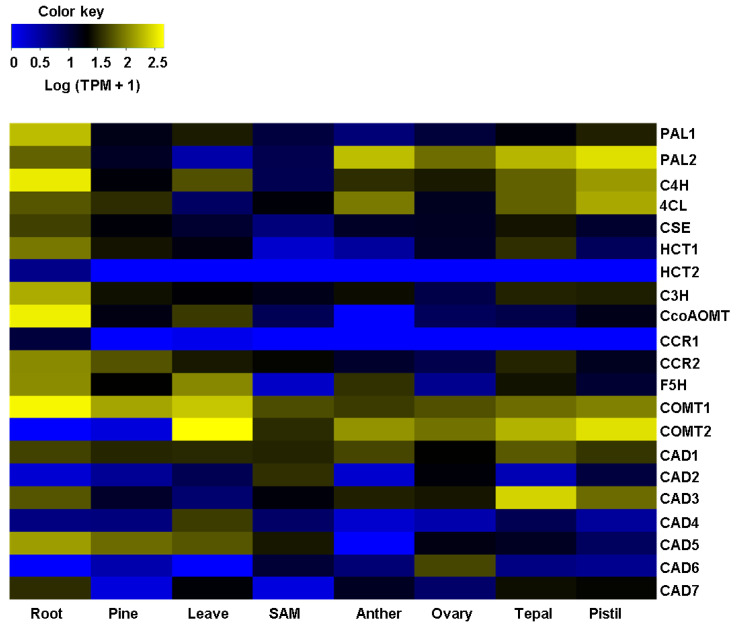
Heatmap of expression profiles of orthologous genes involved with lignin biosynthesis across several tissues/organs of *A. tequilana*. Genes enlisted on the left side are plant biosynthetic genes for lignin according to the literature. *L-Phenylalanine Ammonia-lyase* (*PAL*), *Cinnamic Acid 4-Hydroxylase* (*C4H*), *4-Hydroxycinnamate CoA Ligase* (*4CL*), *Caffeoyl Shikimate Esterase* (*CSE*), *Hydroxycinnamoyl CoA: Shikimate Hydroxycinnamoyl Transferase* (*HCT*), *Coumarate 3-Hydroxylase* (*C3H*), *Caffeoyl CoA 3-O-Methyltransferase* (*CCOAOMT*), *Cinnamoyl CoA Reductase* (*CRR*), *Ferulic Acid/coniferaldehyde 5-Hydroxylase* (*F5H*), *Caffeic Acid 3-O-Methyltransferase* (*COMT*), and *Cinnamyl Alcohol Dehydrogenase* (*CAD*). At the bottom, the last row indicates the RNA-sequencing samples previously reported [16]. SAM: Shoot apical meristem. Hierarchical clustering was conducted on the log (TPM+1). In the color key, yellow denotes high expression, black refers to medium expression, and blue denotes low expression.

Unlike the expression patterns of genes for cellulose formation, a transcriptomic landscape that is more diverse and contrasting was observed for lignin biosynthesis. All the gene isoforms encoding for L-Phenylalanine Ammonia-Lyase2 (PAL2), Cinnamic Acid 4-Hydroxylase (C4H), 4-Hydroxycinnamate CoA Ligase (4CL), Hydroxycinnamoyl CoA: Shikimate Hydroxycinnamoyl Transferase1 (HCT1), and Coumarate 3-Hydroxylase (C3H), enzymes that catalyze shikimate ester intermediates, were found to be from medium to highly expressed in roots and reproductive tissues (Supplementary Figure S2 and Table S3). Expression of *Cinnamoyl CoA Reductase* (*CCR*), a critical gene that drives the first specific step synthesizing the lignin monomers [36], was restricted to two lignified tissues. *AtqCCR1* had a basal expression in roots, while *AtqCCR2* was found to have enhanced expression in roots and pine. Similar to *AtqCCR2*, *Ferulic Acid/Coniferaldehyde 5-Hydroxylase* (*AtqF5H*) was highly expressed in roots, pine, and anthers. Both isoforms for *Caffeic Acid 3-O-Methyltransferase1* and *2* (*AtqCOMT1*, *2*) were widely expressed in almost all the Agave tissues and organs evaluated (Figure 2; Table S3). It is worth mentioning that *AtqCOMT2* was the lignin-related gene with the highest expression level that was found in Agave leaves. With some exceptions, all the *Cinnamyl Alcohol Dehydrogenase* (*CAD*) identified had a discreet expression pattern; *AtqCAD1* had a medium expression level, expressed throughout all the samples analyzed, and *AtqCAD3* had a high expression in tepals (Figure 2; Table S2). *AtqCAD5* was only found in samples with highly lignified tissues, roots, pine, and leaves, suggesting an essential role in monolignol biosynthesis.

### 2.3. Structural and Phylogenetic Characteristics of AtqCESA and AtqCAD Proteins

To analyze in deep of the sets of orthologous genes found, we focused on *CESA* and *CAD*, two genes encoding for key enzymes in determining cellulose and lignin content in plants, respectively, and that were found upregulated in transcriptomes coming from lignocellulose-enriched samples (roots, pine, and leaves). Multiple sequence alignments between all the predicted AtqCESA proteins and *A. thaliana* revealed high sequence conservation, with percentages of identity and similarity ranging from 54–84% and 66–89%, respectively. When these analyses were carried out with *Asparagus officinalis*, the closest phylogenetically monocot species to Agave with publicly available CESA sequences, percentages increased to 44–93% for identity and 54–96% for similarity (Table S3). For CAD proteins, the identity comparison between *A. tequilana* and *A. thaliana* showed 41–75% and 58–81% for similarity. CAD proteins have not been reported for *A. officinalis*; thus, analyses were performed with *O. sativa.* AtqCAD proteins showed 39–80% identity and 52–88% similarity with respect to rice (Table S4).

**Figure 3:**
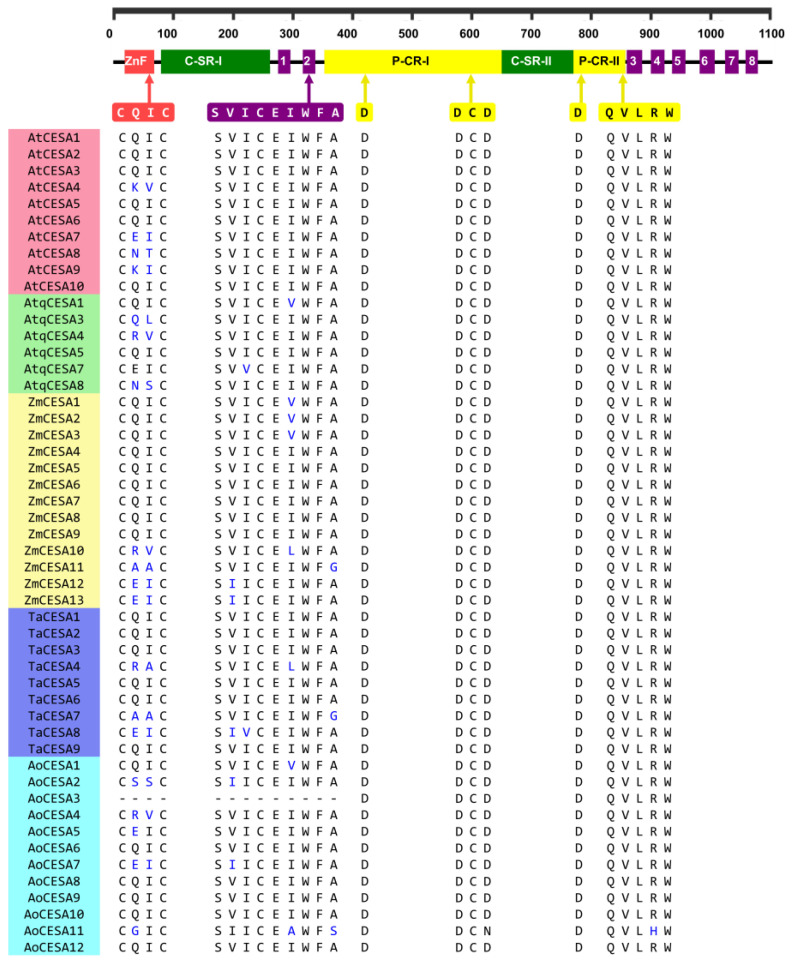
Motif alignment of AtqCESAs with CESAs from other species. *Arabidopsis thaliana* (AtCESA), *Agave tequilana* (AtqCESA), *Zea mays* (ZmCESA), *Triticum aestivum* (TaCESA), and *Asparus officinalis* (AoCESA). Non-conserved residues are highlighted in blue.

Putative AtqCESA proteins are 1020–1090 amino acids (aa) in length, similar in size to those previously reported for other monocots [37]. When multiple aa alignments with CESAs from Arabidopsis, rice, maize, and garden asparagus were carried out, all the specific structural elements reported previously for plant CESA proteins [37,38,39] were found to be highly conserved in the six deduced AtqCESAs (Figure 3). The zinc-finger domain (ZnF), the two high variable class-specific regions (C-SR-I and C-SR-II), and the two plant-conserved regions (P-CR) were fully conserved with respect to Arabidopsis and other monocots included in this analysis (Figure 3). The motif CXXC, located within the ZnF domain and a signature for genuine CESA proteins and useful to differentiate them from CESA-like proteins (CS-L) [37], was also present in all the AtqCESA proteins; in *A. tequilana*, CXXC had two residues C that were invariable in all the sequences analyzed, while the middle residues showed changes (Figure 3). CQ(I/L)C was found in AtqCESA1, AtqCESA3, AtqCESA5, and AtqCESA7, and across all most Arabidopsis and monocot CESAs compared. C(R/K)(V/A)C was found in AtqCESA4 and AoCESA4 (Figure 3), and their corresponding orthologous AtCESA4, ZmCESA10, and TaCESA4. C(N/S)SC was observed only in AtqCESA8 and AoCESA2 (Figure 3). All these variations in the motif CXXC are conservative replacements indeed. For CQ(I/L)C, isoleucine (I) and leucine (L) are isomers, while in C(R/K)(V/A)C and CN/SSC, the replacements occur with aa with equivalent charges in the lateral chain. With respect to the transmembrane helices (TME) that anchor CESA proteins to the plasma membrane, the two amino terminus-located TMEs, TME1 and TME2, and the six TMEs close to the carboxyl terminus, TME3–6, showed slight variation in the AtqCESA sequences analyzed (Figure S3). The full-conserved motif QXXRW, a catalytic site of CESA proteins [40,41,42,43], was identified between the second and third TME. Moreover, the residues D and DCD within P-CR-I and D within P-CR-II, all suggested to be involved in catalyzing long-chain polysaccharides [42], were also found to be full conservatives in all the CESA sequences analyzed (Figure 3).

**Figure 4:**
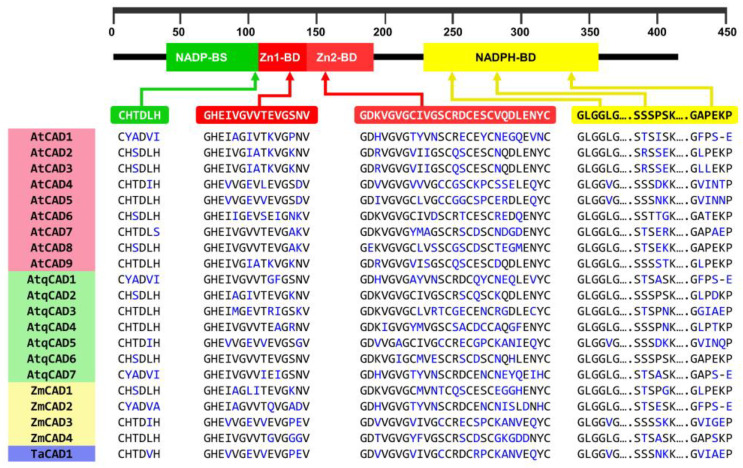
Motif alignment of AtqCADs with CADs from other species. *Arabidopsis thaliana,* (AtCAD) *Agave tequilana* (AtqCAD), *Zea mays* (ZmCAD), and *Triticum aestivum* (TaCAD). Non-conserved residues are highlighted in blue.

The seven *AtqCAD* orthologous predicted for *A. tequilana* encodes for putative proteins with a molecular size ranging from 353 aa to 370 aa. Structural features analysis allowed us to identify the two conserved Zn binding motifs, GHEX2GX5GX2V and GDX9CX2CX2CX7C, previously named Zn1-BD and Zn2-BD, respectively (Figure 4) [44]. The motif Zn1-BD is considered the Zn-catalytic center in bona fide CAD proteins, while Zn2-BD is a structural domain [44,45,46,47]. In AtqCAD proteins, the domains CX4H and the glycine-rich repeat GLGGVG, which participate in binding NADP and NADPH, respectively [5,45], were also identified and were highly conserved compared to all other AtCAD and monocot CADs analyzed (Figure 4). The twelve residues that compose the substrate-binding pocket of CAD proteins, previously reported in rice [48], were conserved or substituted by aa with similar properties (Figure S4). Among all the AtqCADs analyzed, the key phenylalanine (F) residue of bona fide CAD proteins [49,50] was only present in AtqCAD5 at position 299, similar to the well-characterized AtCAD5. Taking all these data together, it is suggested that the deduced AtqCAD proteins belong to the zinc-dependent alcohol dehydrogenases with a potential functional role in the lignin biosynthesis in *A. tequilana*.

**Figure 5:**
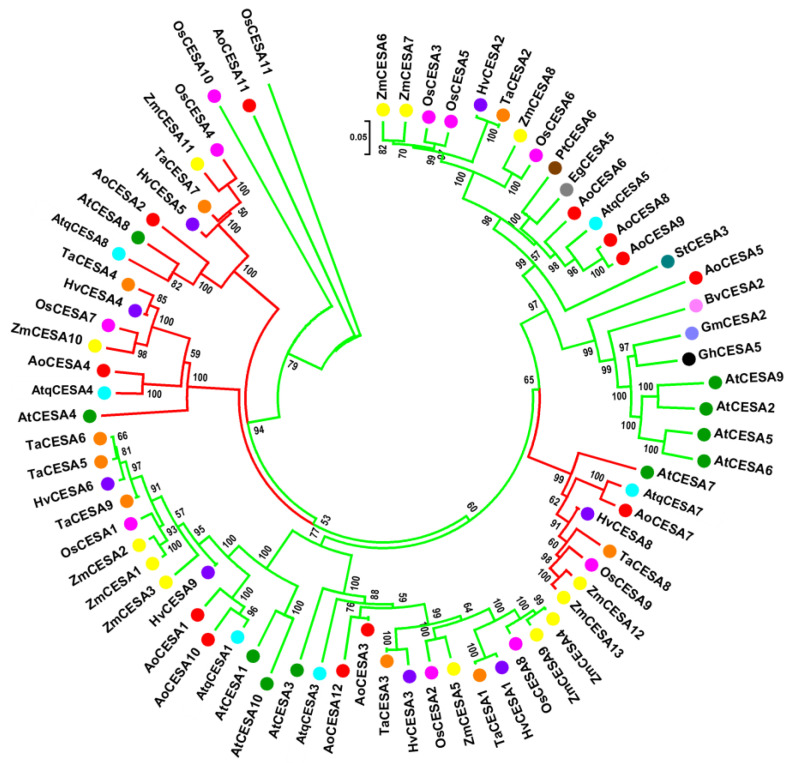
Unrooted phylogenetic tree of the CESAs of *Agave tequilana* (AtqCESA), *Arabidopsis thaliana* (AtCESA), *Beta vulgaris* (BvCESA), *Eucalyptus grandis* (EgCESA), *Glycine max* (GmCESA), *Gossypium hirsutum* (GhCESA), *Hordeum vulgare* (HvCESA), *Oryza sativa* (OsCESA), *Populus trichocarpa* (PtCESA), *Solanum tuberosum* (StCESA), *Asparagus officinalis* (AoCESA), *Triticum aestivum* (TaCESA), and *Zea mays* (ZmCESA). The phylogenetic tree was inferred using a maximum likelihood method.

To gain insight into the evolutionary relationships of putative *CESA* and *CAD* genes of *A. tequilana*, we carried out phylogenetic analysis with all orthologous found in this study and *CESAs* and *CADs* from seven dicots and five monocots, including those well-studied models, such as *A. thaliana*, *A. sativa*, and *Z. mays*. An unrooted tree showed a distribution of all the paralogues *AtqCESAs* in six different clades that grouped closely related orthologous from monocots species, excepting *AtqCESA7*, which was grouped with *AtCESA7* first (Figure 5). This clustering, supported by relatively high bootstrap values, was consistent with the identity and similarity analyses and orthology prediction. Interestingly, three *AtqCESA* genes, *AtqCESA4*, *AtqCESA7*, and *AtqCESA8*, shared clades (red-colored clades, Figure 5) with the well-characterized CESA genes from *A. thaliana* for SCW, *AtCESA4*, *AtCESA7*, and *AtCESA8*, suggesting a similar biological role during cell wall formation in *A. tequilana*.

**Figure 6:**
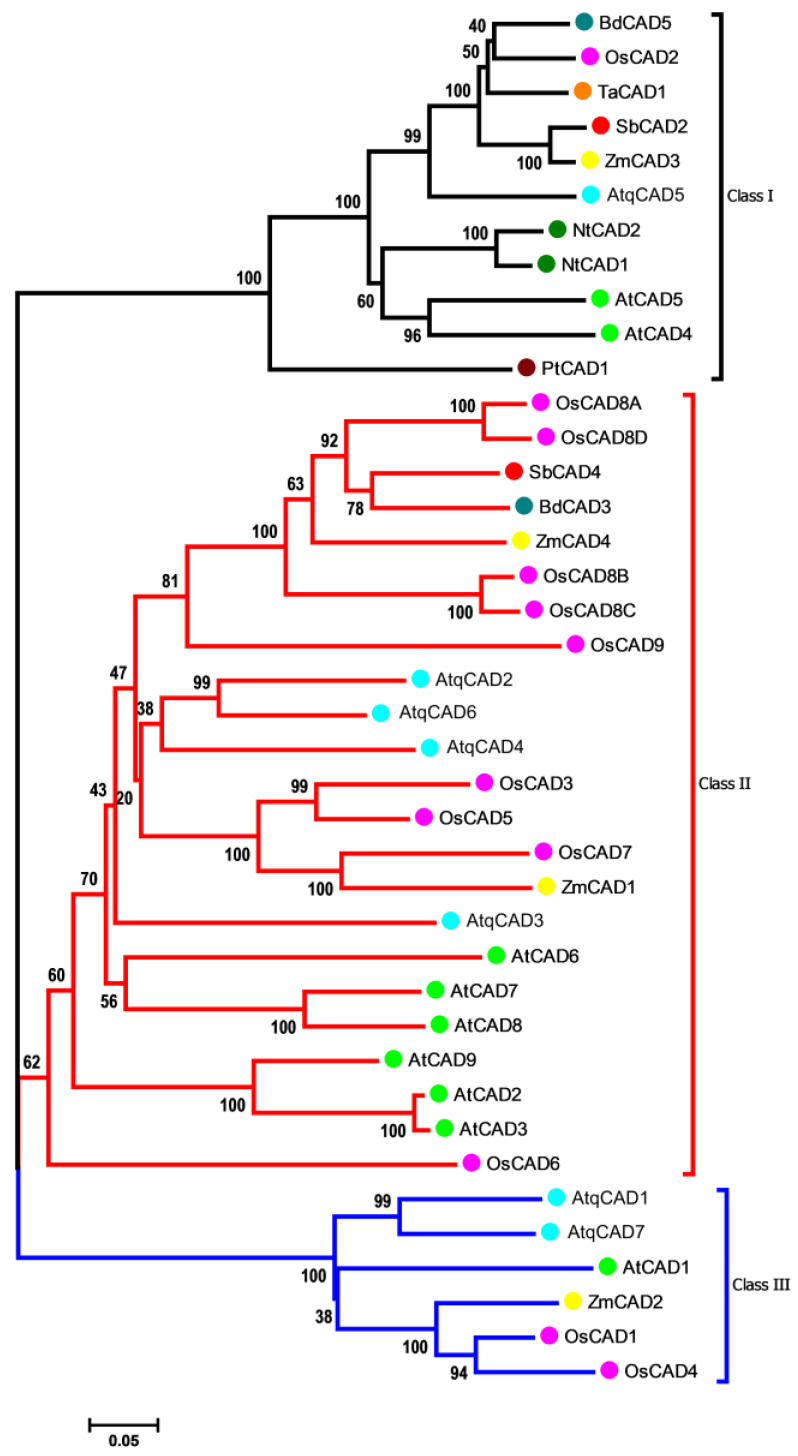
Unrooted phylogenetic tree of the CADs from *Agave tequilana* (AtqCAD), *Zea mays* (ZmCAD), *Oryza sativa* (OsCAD), *Sorghum bicolor* (SbCAD), *Nicotiana tabacum* (NtCAD), *Pinus taeda* (PtCAD), *Brachypodium distachyon* (BdCAD), *Triticum aestivum* (TaCAD), and *Arabidopsis thaliana* (AtCAD).

According to previous phylogenetic studies for monocot *CADs* [44], our analyses for Agave CAD proteins and 32 CADs from other plant species revealed three major classes clearly divided, named Class I, II, and III. CAD Class I can be subdivided into two subgroups (Figure 6). Clade Class I clusters monocot CADs and another for eudicots; this group contains AtqCAD5 and another five members previously considered as bona fide CAD proteins (i.e., those whose products catalyze genuine enzymatic reactions) [44,45,50]. OsCAD2, the closest in silico match with Agave CAD proteins (88% similarity concerning AtqCAD5) (Table S4) was the only rice CAD protein included in this class. Clade CAD class II clusters most of the AtqCADs predicted, AtqCAD2, AtqCAD3, AtqCAD4, and AtqCAD6, and shows no a clear separation for the monocots and eudicots species analyzed. Clade Class III included AtqCAD1 and AtqCAD7 and was the smallest group in the phylogenetic tree obtained (Figure 6).

### 2.4. Retrieved Agave Orthologous Genes for Cellulose and Lignin Biosynthesis Are Preferentially Expressed in Cells Surrounding Leaf Sclerenchyma Fibers

Agave leaves are SCW-enriched organs since their high content of sclerenchyma fibers [51]. Thus, it was expected that SCW-related genes were highly expressed in the leaf transcriptome mined here, although leaf transcriptomic heatmaps showed null, low, and medium expression levels of key genes for cellulose and lignin metabolism. We suppose these expression levels could be underrated because of sample-processing issues during total RNA isolation. During Agave sample processing, fibers are eliminated usually when leaf samples are grounded in liquid nitrogen, throwing out cells associated with fibers or developing fibers that potentially express biosynthetic genes for SCW components. In this last part of our study, we tested this hypothesis by quantifying the expression levels of some Agave genes using qPCR, predicted to be working in biosynthetic pathways for cellulose or lignin during SCWs formation, according to our orthology inference, transcriptomic data clustering, structural motif, and phylogeny analysis.

**Figure 7:**
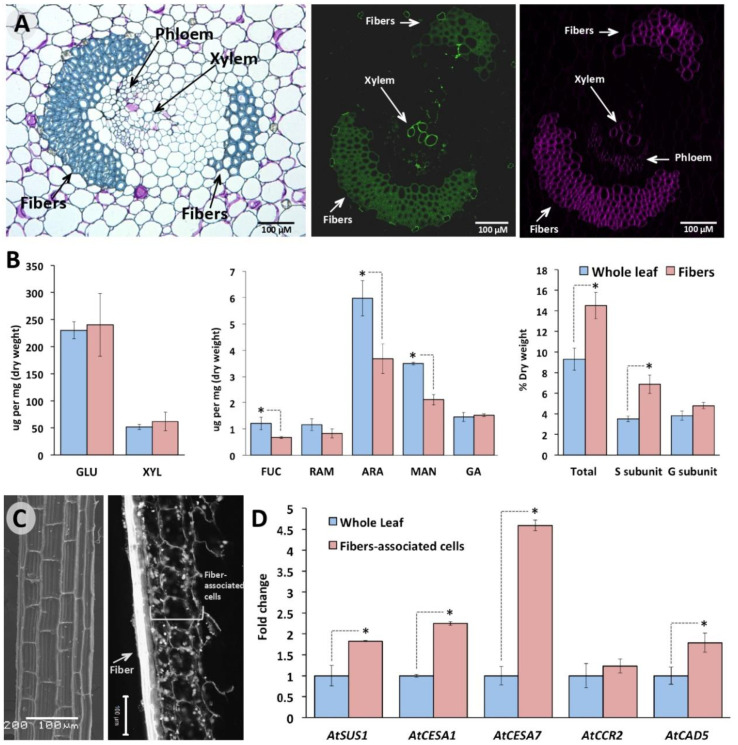
Key genes for cellulose and lignin have a high expression in cells surrounding fibers of A. tequilana leaves. (**A**) Sclerenchyma fibers surrounding vascular bundles. Heavily thickened secondary cells forming fiber caps walls were visualized in cross leaf sections after Toluidine Blue O (TBO) staining (left); lignification of fibers and xylem cells were visualized by lignin autofluorescence imaged by confocal laser scanning microscopy (LCSM) (middle). Cellulose was detected by Direct Red 23 staining and visualized by LCSM (right). (**B**) Monosaccharides analysis (left and middle) by high-performance anion exchange chromatography with pulsed amperometric detection (HPAEC-PAD) and lignin quantification (left) by pyrolysis gas chromatography/mass spectrometry (py-MBMS) of sclerenchyma fibers and cell walls of whole leaf. FUC: Fuccose, RAM: ramose, ARA: arabinose, MAN: mannose, GA: galacturonic acid, GLU: glucose; XYL: xylose. (**C**) Scanning Electronic Microscopy (SEM) of fiber (left) and fibers-associated cells stained with propidium iodide and imaged by LSCM (right). (**D**) Expression levels of key genes for cellulose and lignin biosynthesis quantified by qPCR assays. Total RNAs isolated from whole leaf and fibers-associated cells were used for RT-qPCR assays (right). Glycerol-3-Phosphate Dehydrogenase (GPDH) was used as a load gene. Values are means ± SD. Asterisks indicate significant statistical differences between fibers/fibers-associated cells and whole leaf determined by Tukey’s HSD test (*p* ≤ 0.05).

Histology analysis of cross sections showed typical sclerenchyma fibers surrounding vascular bundles in Agave leaves, which according to their position with respect to xylem can be classified as extraxylary fibers [51] (Figure 7A, left). An enhanced cellulose and lignin deposition was detected in the cell walls of these extraxylary fibers compared to those of surrounding parenchyma tissue (Figure 7A, middle and right). These observations were confirmed by monomers quantification of cellulose, hemicellulose, and lignin (Figure 7B). Although no statistical significance was found between cellulose levels fibers and whole leaf, determined by quantifying their glucose subunits, a tendency of higher content was observed in fibers. When hemicellulose components were measured, parenchyma cell walls had a higher content for all the monosaccharides compared with fibers, except gluconic acid (GA) (Figure 7B, middle). On the other hand, lignin analysis revealed that total lignin content is about 1.6-fold higher in fibers compared to that of surrounding parenchyma, mostly explained by higher levels of syringyl (H) monolignols in the sclerenchymatous tissue (Figure 7B, left), in line with that known for fibers [52]. To circumvent the potential bias described above in the SCW-involved gene expression analysis, we contrasted the gene expression patterns *AtqSUS2*, *AtqCESA1*, *AtqCESA7*, *AtqCCR2,* and *AtqCAD5* between cells associated to leaf fibers (Figure 7C, left) and whole leaf. All the evaluated genes were more expressed in the fibers-associated cells than the in whole leaf, except for *AtqCCR2*. Among the three genes analyzed for cellulose biosynthesis, *AtqCESA7*, which we predicted to be the orthologous for *AtCESA7* that is widely known for its vital role during CSW biogenesis, was the highest expressed in cells surrounding fibers, with a 4.5-fold change respect to the levels found in whole leaf (Figure 7C, right). Similarly, *AtqCAD5*, the Agave CAD isoform clustered in our phylogenetic analysis with bona fide CAD proteins, had an expression level almost 2-fold higher in the fiber-associated cells than in the whole leaf (Figure 7C, right). Overall, this upregulation of predicted genes involved with SCW biogenesis in cells associated with extraxylary fibers is consistent with the chemical composition of such tissues.

## 3. Discussion

Unlike in dicots, biosynthetic and regulatory genes involved with cell wall metabolism are less known in monocots. Although significant advances have been achieved for maize, wheat, and rice, for emerging bioenergy crops such as Agave, our knowledge about cell wall biogenesis is still very limited. For monocot species, in the absence of reference nuclear genomes, transcriptome resources have provided insights into the biosynthesis and regulatory mechanisms for cellulose and lignin biosynthesis, two key components for cell walls.

Although the biosynthetic pathways for cellulose and lignin have been explored in * A. sisalana*, *A. fourcroydes*, and the hybrid H11648 [17,19], our study dissects both routes in *A. tequilana* for the first time, and provides their transcriptomic landscapes in contrasting tissues and organs for gaining more insights into the biological significance of the predicted orthologous. Variation in the numbers of isoforms found in both metabolic routes between our results and earlier analyses may reflect species or tissue-specific expression patterns, expressed according to the physiological status, development, and environment tested, as reported previously in Agave species [12,19,53]. For example, in silico expression profiling and qPCR assays of genes of the glycoside hydrolase family 32 revealed new vacuolar invertase isoforms only for *A. tequilana* and *A. deserti* but not for *A. victoriae-reginae*, and *A. striata* [12]. Immunolocalization of mayahuelin, a type I ribosome-inactivating protein (RIP), showed differential protein abundance, including absence, depending on the Agave species or cultivar screened [53]. Several studies support the idea that *CESA* genes belong large families in monocots. In wheat, maize, and rice, 14, 10, and 11 CESAs members have identified, respectively [37,54,55,56]. In grasses, in *Panicum virgatum* 14 *CESA* genes have been reported [56], while an RNA-seq analysis revealed 27 *CESA* genes expressed during stem development of *Pennisetum purpureum* [57]. Here, we retrieved only six *CESA* orthologous in *A. tequilana*, which is quite comparable with that found in the hybrid H11648 (five CESA genes) [17] but contrasts with that reported recently in *A. fourcroydes*, *A. sisalana*, and H11648 (14 CESA genes) [19].

*For COMT* genes, which drive the first specific step conducting lignin syringyl unit biosynthesis, Agave species seem to harbor small families. We found only two putative *AtqCOMT* genes in *A. tequilana,* while three genes have been identified in *A. fourcroydes*, *A. sisalana*, and the hybrid H11648 [19], findings similar to that found in maize [58] but different from the 18 genuine COMT and COMT-like sequences found in sugarcane transcriptomes [59]. For *CAD* genes, the existence of multiple orthologous found in *A. tequilana* is also consistent is a high number *CAD* genes reported in fiber-producing Agave species [19], and other monocots, such as rice (12 genes) and *Setaria viridens* (11 genes) [60,61]. For the rest of cellulose and phenylpropanoid pathway genes, they were grouped in small families or even classified as unigenes, comparable with that reported in *A. fourcroydes*, *A. sisalana*, and the hybrid H11648 [17,19]. This fact, and considering that these two Agave species show that different ploidy levels ranging from 2n to 5n [62,63,64] support the previous suggestion of null existence of expanded gene families in Agaves [9], at least for lignin metabolism. Among the genes identified in our study, it is worth mentioning the finding of *CSE*, that encodes an enzyme that converts caffeoyl CoA to caffeic acid [65], previously also reported in transcriptomes of fiber-producing Agaves [19]. The existence of *CSE* genes expressed in diverse Agavaceae members allows us to speculate this gene has a functional role in this taxonomic family; however, its real participation in lignin biosynthesis in plants is still controversial as *CSE* is absent in many monocots genomes, including *B. distachyon*, *S. italica*, *S. bicolor*, *H. vulgare*, *Oriza sativa*, and *Z. mays* [66].

Interestingly, our analysis also revealed that orthologous predicted for genes located upstream of lignin-specific steps but still into the general phenypropanoid pathway have isoforms highly expressed in reproductive tissues of *A. tequilana*. It is known that CCR and CAD-upstream enzymes provide intermediates not only for lignin biosynthesis but also for hydroxycinnamoyl esters, isoflavones, and coumarin derivatives [67,68]. Recently, it was shown that genes of the phenylpropanoid pathway, including lignin-specific biosynthetic genes, are expressed in the tapetum in Arabidopsis [69]. In line with this, in *A. tequilana*, all these genes are also highly expressed in anther, ovary, tepals, and pistil. We found enhanced expression for some isoforms of *AtqPAL*, *AtqC4H*, *Atq4CL*, *AtqCOMT*, and *AtqCAD*, which may yield phenylpropanoid derivaties; indeed, a high content of kaempferol glycosides has been quantified in pollen and flowers of *A. americana* and *A. durangneisis*, respectively [70,71]. Slight transcriptional changes for *AtqHCT1* and *AtqC3H* observed in *A. tequilana tepal* and pistil could explain the previously reported chlorogenin levels of Agave flowers [70]. In Agave plants, all these phenolic compounds may prove UV protection to reproductive tissues, as assumed in Arabidopsis [69], and potential protection against pathogens.

Our detailed analyses of key enzymes for cellulose and lignin, *CESA* and *CAD*, respectively, gave some clues about in their participation in cell wall biosynthesis. Protein sequences analysis was consistent with phylogenetic clustering obtained for both types of enzymes, suggesting a high evolutionary conservation within monocots and with respect to dicots. Identification of all the structural and functional motifs of typical plant CESA proteins in all the *AtqCESAs* predicted here indicate we retrieved full-length *CESA* sequences, encoding genuine CESA proteins, and potentially involved in UDP-glucose monomers polymerization. Although we were unable to distinguish a clear conservation of the new motifs proposed to classify monocot CESA into PCW CESAs or SCW CESAs [37], the phylogenetic analysis and transcriptional profiling of these *AtqCESAs* in tissues and organs with contrasting development level suggest *AtqCESA1*, *AtqCESA3*, and *AtqCESA4* are putative PCW *AtqCESAs*, while *AtqCESA5* and *AtqCESA7* are SCW *AtqCESAs*. *AtqCESA8*, in spite of its orthologous in *A. thaliana* (*AtCesA8/IRX1*), is an SCW CESA [72] that was found to be expressed relatively uniform across all the transcriptomes mined, giving a unclear idea about their role in cell metabolism. Among the *AtqCADs* identified, *AtqCAD5* is the phylogenetic closest to bona fide CAD proteins previously reported in monocots. This finding suggests that *AtqCAD5* is a bona fide CAD protein in *A. tequilana*. This is also supported by another evidence line: high expression in parenchyma cells associated to extraxylar fibers.

Molecular mechanisms involved in cell wall formation of sclerenchyma fibers are little known in plants, especially for extraxylar fibers (leaf fibers), as leaf fibers are classified in Agave [51,73]. From that is known of interfascicular fibers in *A. thaliana*, it is assumed that fiber lignification is a cell autonomous process because fibers necessary require cell death to achieve their structural support function [52,74]. However, leaf fibers have a different ontogeny compared to interfascicular fibers [74,75]; thus, events leading to cellulose and lignin formations are remained to be uncovered fully in monocots. Although our dissection technique of fibers is not enough precise to discard contamination of xylem elements, our qPCR analysis for some key genes of cellulose and lignin clearly showed an enhanced transcriptional expression in tissue surrounding to fibers and vascular bundles, suggesting: (a) *AtqCESA7* and *AtqCAD5* are enzymes working in SCW formation, and (b) cooperative or partial cooperative lignification is underlying leaf fiber development in *A. tequilana*. Agave genome sequencing will help to know with accuracy the gene families’ size and the evolution history of genes related to cell wall metabolism. Likewise, further research will be required to uncover the molecular mechanisms underlying fiber development in Agave species, which seem to be an excellent model plant to study sclerenchyma tissue.

## 4. Materials and Methods

### 4.1. Data Mining for Cell Wall-Related Genes

To identify genes involved with cellulose and lignin biosynthesis (Figures S1 and S2, respectively), we mined data of eight transcriptomes from roots, stem apical meristem, and juvenile and adult leaves of *A. tequilana*, sequenced by HiSeq 2000 (Ilumina) and previously reported [9] (GenBank SRP019885; Table S5). Raw data of all these short-read cDNA libraries were used for a novo transcriptome assembly and functional annotation. Before assembly, quality control of sequenced reads was carried out. Raw reads were filtered for high-quality sequences using the qualityControl.py.script from Next-Generation Sequencing (NGS) toolkits (https://github.com/Czh3/NGSTools/blob/master/qualityControl.py, accessed on 30 August 2021) with the default settings. Overlapping reads were merged into a single longer read using Seqprep program (https://github.com/jstjohn/SeqPrep, accessed on 5 September 2021). The resulting reads were used for assembly by Trinity (V2.4.0) [76], and the unigenes obtained were trimmed from polyA/T tails, ends rich in undetermined bases, and low-complexity sequences by using SeqClean software (http://compbio.dfci.harvard.edu/tgi/, accessed on 10 September 2021). Frameshift errors were corrected in the predicted open-reading frames by AlignWise pipeline [77]. Later, a non-redundant dataset was generated using the BLASTClust program (https://www.ncbi.nlm.gov/Web/Newsltr/Spring04/blastlab.html, accessed on 12 September 2021). Finally, the coding regions corresponding to the unigenes generated were translated to proteins. Using the BBH (Best BLAST Hit) method, homologous proteins were identified to proteins translated from A. tequilana. As reference proteins, we used complete genomes sequenced of five different species plants, which were strategically selected as representatives of specific clades along the phylogenetic distribution of angiosperm plants: *Vitis vinifera* within the order Vitals, *Amborella trichopoda* within Amborellaceae, *S. lycopersicum* within Solanales, *Z. mays* within Poales, and *A. thaliana* within Brassicales.

### 4.2. Orthologous Groups Identification and Protein Subcellular Localization Prediction

The closest orthologous and paralogous genes were identified using the Markov clustering algorithm [78], an inflation value of 1.5 [79], and the OrthoMCL software v. 2.0.9 [80]. For the formation of the orthologous groups, a minimum length of 30 aa was considered in all the proteins included in the analysis. Predicted proteins were analyzed for subcellular localization using the WoLF PSORT tool available in www.wolfpsort.hgc.pj (accessed on 14 September 2021).

### 4.3. Sequence Alignment and Phylogenetics of AtqCESA and AtqCAD Families

To identify structural and functional motifs, multiple alignments of AtqCESA and AtqCAD protein sequences were performed using ClustalW with default parameters [81]. Multi-species phylogenetic trees for both types of proteins were then constructed using MEGA 6.06 [82] with the Neighbor-joining method (1000 boot-strap trials, the Poisson model) based on the previous multi-alignments. CESA and CAD protein sequences of *Z. mays*, *O. sativa*, *A. officinalis*, *A. thaliana*, *Populus trichocarpa*, *T. aestivum*, *B. vulgaris*, *Eucalyptus grandis*, *G. max*, *Gossypium hirsutum*, *H. vulgare*, and *S. tuberosum*, included in the phylogenetic analyses, were obtained from the UniProt database (www.uniprot.org/help/uniprotkb). Identity/similarity analysis was carried out for all the CESA and CAD proteins identified in *A. tequilana*. For this, pairwise determinations were calculated with Sequence Manipulation Suite (SMS), available at https://www.bioinformatics.org/sms2/ident_sim.html (accessed on 18 September 2021) [83].

### 4.4. In Silico Gene Expression Analysis of Cellulose and Lignin Biosynthesis

Our approach to propose robust sets of closest orthologous genes encoding enzymes for cellulose and lignin was carried out an expression profiling analysis after orthology prediction, using data of eight RNA-seq transcriptomes [16] [June Simpson lab. Unpublished data] obtained from a wide range of *A. tequilana* tissues and organs, enriched with PCWs (meristem, anthers, ovules, tepals, and pistils) and SCWs (roots, stems, and leaves). Normalized data of these RNAseq transcriptomes were used to get tissue expression profiles using RSEM software [84]. RSEM uses an expectation-maximization algorithm, which produces an array with the expression profiles in transcripts per million (TPM). Heatmaps were created based on the expression levels using the heatmap.2 function of the gplots library of the statistical package R version 3.6.1 (https://www.r-project.org) (accessed on 21 September 2021).

### 4.5. Samples Collection and Processing

For lignin and structural carbohydrate quantifications and gene expression analysis, *A. tequilana* leaves samples were collected and processed as described immediately. Adult leaves (third full-expanded leaf) were collected from 4-year plants in Rancho Agropecuaria Santa Genóveva, Campeche México (19°33′03.0″ N 90°01′10.9″ W). On the lab-bench, three types of samples were obtained from leaves: pooled samples of the whole leaf, cells surrounding fibers, and fibers. For pooled samples of the whole leaf, three fractions (16 cm^2^) were dissected from the basal (C), medium (B), and distal (A) parts, frozen with liquid nitrogen (N_2_), and kept at −80 °C until processing. These three leaf fractions were used to get a landscape of the whole leaf, as reported previously in Agaves [9]. For isolation of cells surrounding fibers, leaves were longitudinally segmented with a sterilized knife, and then fibers were detached one by one and immediately frozen with N_2_ and kept at −80 °C until processing. The presence of surrounding cells still to fibers was verified by propidium iodide staining (0.2 μg ul^−1^) (Sigma-Aldrich, St. Louis, MO, USA) and visualized by laser scanning confocal microscopy (514 nm line for excitation, Carl Zeiss, Jena, Germany). Fibers isolated after grounding with N_2_ were washed deeply with tap water and dried to 65 °C.

### 4.6. Monosaccharide and Lignin Composition

Monosaccharide and lignin profiles were quantified in whole leaves and dried fibers. The leaf fractions A, B, and C, isolated as described above, were grounded separately using N_2_ and then lyophilized. A pooled sample representing the whole leaf was prepared using equal amounts of these fractions. Whole leaves and dried fibers were then analyzed by triplicate using high-performance anion exchange chromatography with a pulsed amperometric detector (HPAEC-PAD). Starch-free cell walls from whole leaf and fibers were prepared as reported [85,86]. Hydrolysates were diluted with deionized water and then filtered through a nylon membrane of 0.22 mm before injection to a Dionex ICS-3000 (Thermo, Waltham, MA, USA) equipped with a CarboPac PA-100 guard column (4 mm × 50 mm) and a CarboPac PA-100 analytical column (4 mm × 250 mm) (Thermo, Waltham, MA, USA). Parameters used for monosaccharide separation were those previously reported [87]. Lignin composition (total content and monomers) was determined by pyrolysis gas chromatography/mass spectrometry (py-MBMS) as previously reported [86,88]. Briefly, processed samples were pyrolyzed (PY-2020 iS, Frontier Labs) for 2 min at 500 °C to produce volatile compounds. NIST8492 (*P. deltoids*) and aspen (*P. tremuloides*) analytical standards were included in each run of the experiment for quality control. Volatile compounds were analyzed for lignin using the MBMS (Extrel Max-1000, Super-Sonic, Pittsburgh, PA, USA), and raw data were processed through UnscramberX 10.1 software. 

### 4.7. Histochemistry and Microscopy

For histochemical detection of lignin and cellulose associated with extraxylary fibers, samples of the leaf fraction C described above were processed as reported [86]. Chemical-fixed samples in 4% paraformaldehyde (*w/v*) (Sigma-Aldrich, St. Louis, MO, USA) were dehydrated in ethanol series and then embedded in JB4- water-soluble resin (Electron Microscopy Sciences, Hatfield, PA, USA). Cross sections, obtained with a Leica EM UC7 ultramicrotome (Leica Microsystems, Wetzlar, Germany), were used for histochemical analyses. Cellulose was detected by staining with 0.1% Direct Red 23 (Sigma-Aldrich, St. Louis, MO, USA) and imaged with a Leica SP8-iPhox inverted microscope (excitation 562 nm; emission 580–615 nm) (Leica Microsystems, Wetzlar, Germany), while lignin was detected by autofluorescence. Blue or blue-green-stained extraxylary fibers and xylem vessels were revealed using a 0.02% Toluidine Blue O solution (Sigma-Aldrich, St. Louis, MO, USA). For SEM analysis, dry fibers were sputter-coated with a gold layer and visualized using a JEOL JSM-6360LV (Tokyo, Japan).

### 4.8. RT-qPCR Analyses

For gene expression analysis, reverse transcription–quantitative real-time PCR (RT-qPCR) assays were carried out evaluating two kinds of samples: whole leaf and cells surrounding fibers. Ultra-frozen leaf fractions A, B, and C, obtained as described above, were processed separately for RNA isolation. Fibers with cells attached were deeply grounded in N_2_, then the fibers were removed, and the tissue left on the mortar was used for further analysis. Extraction and purification of total RNA were carried out following the manufacturer’s instructions (QIazol 79306, RNeasy MinElute Cleanup kit 74204, QIAGEN, Germantown, MD, USA). For the whole leaf, we prepared a pooled sample using equal amounts of total RNAs from fractions A, B, and C. Three biological replicates were processed for each type of sample, and five minipreps for each biological replicate. cDNA templates were synthetized using 1.7 μg of purified total RNA, oligodT primers (10 μM), and SuperScript III (18080093, Invitrogen/TermoFisher, Waltham, MA, USA). For real-time quantitative PCR (qPCR) analysis, based on the in silico expression patterns of the transcriptomes analyzed, we selected some key biosynthetic genes for cellulose (AtqSUS2, AtqCESA1, and AtqCESA7) and lignin (AtqCCR2 and AtqCAD5) to quantify their expression levels. Gene-specific oligonucleotides used in this study are listed in Table S6. qPCR reactions (20 µL) contained: 8 µL de SYBR Green PCR Master Mix (Applied Biosystems, Foster City, CA, USA), 1.5 µL of template cDNA (cDNA diluted 1: 10), 2 μL of the mix of specific oligonucleotides (1 μL of each one, at 10 μM), and 8.5 μL of ultrapure water (Invitrogen/Life Technologies, Waltham, MA, USA). Reactions were run in a StepOne Real-Time PCR System equipped with StepOne Software v2.3 (Applied Biosystems, Foster City, CA, USA). Cycling conditions were as follows: one cycle at 95 °C for 10 min, 38 cycles of 95 °C for 15 s, and one cycle at 60 °C for 1 min, linked to a default dissociation stage program to detect non-specific amplification. *GLYCERALDEHYDE-3-PHOSPHATE DEHYDROGENASE* (*GAPDH*) was used as the internal reference gene [89], and relative expression levels were determined using the comparative quantification method 2^−ΔΔCt^ [90].

## 5. Conclusions

Our results identified sets of orthologous for biosynthetic genes for cellulose and lignin pathways in *A. tequilana*. Sequence analyses and phylogeny reflected a high evolutionary conservation between all the Agave isoforms retrieved, and those from monocots and eudicots included in this study, which could have biological roles in cell wall biogenesis and metabolism as suggested by their differential expression patterns found. In sclerenchyma fiber development, the high expression of AtqCAD5 in parenchyma cells associated to extraxylary fibers suggest the existence in Agave of a cooperative lignification mechanism similar to that known for xylem tissue.

## Figures and Tables

**Table 1:** Detailed sequence information of genes involved in cellulose biosynthesis in *Agave tequilana*.

Gene	ORF (nt)	Protein (aa)	Calculated Mw (Da)	Theoretical pI	Predicted Subcellular Location and Score *
*AtqSPS1*	3168	1055	118,083.33	5.77	nucl: 5, cyto: 4, chlo: 3
*AtqSPS2*	3252	1083	120,102.55	6.11	nucl: 5, cyto: 5, chlo: 2
*AtqSPP1*	1275	424	47,733.35	5.54	cyto: 5, nucl: 4, chlo: 2
*AtqSPP2*	1275	424	48,024.71	6.00	chlo: 7, nucl: 3, mito: 3
*AtqFK1*	651	216	23,897.62	5.62	chlo: 6, cyto: 5, cysk_nucl: 2
*AtqFK2*	969	322	34,489.54	5.20	cyto: 8, chlo: 3, pero: 2
*AtqFK3*	996	331	35,476.84	5.03	pero: 11, chlo: 1, nucl: 1
*AtqFK4*	1224	407	43,435.34	6.13	chlo: 14
*AtqFK5*	1107	368	39,509.00	5.50	cysk: 9, cyto: 3, chlo: 1
*AtqGPI1*	1521	506	55,292.25	4.97	cyto: 9, chlo: 3, mito: 1
*AtqPGM1*	1872	623	68,121.76	6.82	chlo: 14
*AtqPGM2*	1749	582	63,450.95	6.02	cyto: 11, chlo: 2, nucl: 1
*AtqSUS1*	2532	843	96,280.19	8.07	cyto: 12, chlo: 1, plas: 1
*AtqSUS2*	2448	815	92,525.91	6.03	cyto: 9, chlo: 2, nucl: 1
*AtqSUS3*	2424	807	92,436.65	5.83	cyto: 5, mito: 4, chlo: 3
*AtqHXK1*	1488	495	53,179.08	6.07	chlo: 13, mito: 1
*AtqHXK2*	1497	498	54,454.50	5.87	chlo: 11, nucl: 1, extr: 1
*AtqUGP1*	1422	473	51,920.96	6.09	cyto: 11, chlo: 2, E.R.: 1
*AtqUGP2*	1575	524	58,194.63	7.74	chlo: 7, mito: 6, nucl: 1
*AtqUGP3*	2262	853	93,824.45	6.52	chlo: 10, mito: 2, nucl: 1.5
*AtqCESA1*	3264	1087	122,300.01	6.46	plas: 13, E.R.: 1
*AtqCESA3*	3180	1059	119,098.30	8.14	plas: 10, nucl: 2, mito: 1
*AtqCESA4*	3174	1057	120,445.31	6.90	plas: 11, nucl: 2, E.R.: 1
*AtqCESA5*	3273	1090	122,477.03	6.85	plas: 13, E.R.: 1
*AtqCESA7*	3153	1058	118,824.62	6.34	plas: 12, nucl: 1, E.R.: 1
*AtqCESA8*	3063	1020	114,790.86	6.57	plas: 12, nucl: 1, vacu: 1

Sucrose Phosphate Phosphatase (SPP), Sucrose Phosphate Synthase (SPS), Fructokinase (FK), Glucose-6-PhosphateIisomerase (GPI), Phosphoglucomutase (PGM), Sucrose Synthase (SUS), Hexokinase (HXK), UDP-Glucose Pyrophosphorylase (UGP), and Cellulose Synthase (CESA). Molecular weight (Mw), pI: Isoelectric point (pI). Chloroplast or thylakoid lumen (clo), cytoskeleton (cysk), cytosol (cyto), endoplasmic reticulum (E.R.), extracellular or cell wall (extra), mitochondrion (mito), nucleus (nucl), peroxisome (pero), plasma membrane (plas), vacuolar membrane (vacu). Localization names joining two abbreviations indicate dual localization. * The score means the number of nearest neighbors with the same subcellular localization indicated. Only the three highest scores are shown.

**Table 2:** Detailed sequence information of genes involved in lignin biosynthesis in *Agave tequilana*.

Gene	ORF (nt)	Protein Size (aa)	Calculated Mw (Da)	Theoretical pI	Predicted Subcellular Location and Score *
*AtqPAL1*	2103	700	75,908.60	5.60	chlo: 4, plas: 3, cyto: 2
*AtqPAL2*	2112	703	75,997.81	5.92	chlo: 4, plas: 3, cyto: 2
*AtqC4H*	1518	505	57,804.27	9.19	plas: 10, E.R.: 3, vacu: 1
*Atq4CL*	1689	562	59,781.88	5.52	E.R.: 5.5, E.R._plas: 3.5, cyto: 3
*AtqCSE*	990	329	36,767.97	6.09	nucl: 4, cyto: 4, mito: 2,
*AtqHCT1*	1326	441	48,196.19	6.22	cyto: 7, chlo: 4, nucl: 2
*AtqHCT2*	1302	433	47,684.51	6.02	cyto: 10, chlo: 3, mito: 1
*AtqC3H*	1539	512	58,118.34	7.23	chlo: 13, mito: 1
*AtqCCoAOMT*	771	256	28,666.75	5.30	cyto: 7, chlo: 3, cysk: 2
*AtqCCR1*	981	326	35,680.92	6.18	chlo: 6, plas: 3, vacu: 2
*AtqCCR2*	1017	338	36,883.26	5.73	chlo: 6, plas: 4, vacu: 2
*AtqF5H*	1545	514	57,197.12	6.81	chlo: 5, plas: 4, nucl: 2.5
*AtqCOMT1*	1092	363	39,707.87	5.58	cyto: 9, chlo: 2, cysk: 2
*AtqCOMT2*	1095	364	39,372.20	4.76	cyto: 10, cysk: 2, chlo: 1
*AtqCAD1*	1062	353	38,482.99	7.05	cysk: 8, extr: 3, chlo: 1
*AtqCAD2*	1107	368	39,485.53	6.75	cysk: 11, cyto: 2, pero: 1
*AtqCAD3*	1071	356	38,497.48	6.16	cyto: 8, cysk: 4, plas: 1
*AtqCAD4*	1065	354	37,854.72	6.86	cyto: 11, cysk: 2, plas: 1
*AtqCAD5*	1074	357	38,286.33	5.82	cyto: 10, mito: 1, plas: 1
*AtqCAD6*	1113	370	39,936.18	6.60	cyto: 4, chlo: 3, nucl: 2
*AtqCAD7*	1065	354	38,779.55	7.55	cysk: 9, cyto: 2, extr: 2

L-Phenylalanine Ammonia-Lyase (PAL), Cinnamic Acid 4-Hydroxylase (C4H), 4-Hydroxycinnamate CoA Ligase (4CL), Caffeoyl Shikimate Esterase (CSE), Hydroxycinnamoyl CoA: Shikimate Hydroxycinnamoyl Transferase (HCT), Coumarate 3-Hydroxylase (C3H), Caffeoyl CoA 3-O-Methyltransferase (CCoAOMT), Cinnamoyl CoA Reductase (CRR), Ferulic Acid/Coniferaldehyde 5-Hydroxylase (F5H), Caffeic Acid 3-O-Methyltransferase (COMT), and Cinnamyl Alcohol Dehydrogenase (CAD). Molecular weight (Mw), pI: Isoelectric point (pI). Chloroplast or thylakoid lumen (clo), cytoskeleton (cysk), cytosol (cyto), endoplasmic reticulum (E.R.), extracellular or cell wall (extra), mitochondrion (mito), nucleus (nucl), peroxisome (pero), plasma membrane (plas), vacuolar membrane (vacu). Localization names joining two abbreviations indicate dual localization. * The score means the number of nearest neighbors with the same subcellular localization indicated. Only the three highest scores are shown.

## Data Availability

All data generated or analyzed during this study are included in this published article and its Supplementary Information Files.

## Supplementary Materials

The following supporting information can be downloaded at: https://www.mdpi.com/article/10.3390/plants11111496/s1, Figure S1: Biosynthetic pathway for cellulose in plants; Figure S2: Biosynthetic pathway for lignin in plants; Figure S3: AtqCESA proteins have conserved transmembrane domains; Figure S4: Protein alignment for AtqCAD; Table S1: Expression levels of genes involved in cellulose biosynthesis in *Agave tequilana* in several organs and tissues sampled; Table S2: Expression levels of genes involved in lignin biosynthesis in *Agave tequilana* in several organs and tissues sampled; Table S3: Percentages of identity/similarity between amino acid sequences of orthologous *CESA* genes of *A. tequilana* (*AtqCESA*), *A. officinalis* (*AoCESA*), and *A. thaliana* (*AtCESA*); Table S4: Percentages of identity/similarity between amino acid sequences of orthologous *CAD* genes of *A. tequilana* (*AtqCAD*), *O. sativa* (*OsCAD*), and *A. thaliana* (*AtCAD*); Table S5: cDNA libraries used in this study, previously reported by Gross et al., 2013 and deposited in Genbank (SRP019885); Table S6: Gene-specific oligonucleotides used for qPCR analysis. Supplementary Materials: DNA sequences for Atq orthologous for cellulose and lignin biosynthesis.
