# Supplementary material for: Transcriptome Mining Provides Insights into Cell Wall Metabolism and Fiber Lignification in Agave tequilana Weber
Source: Plants (Basel). 2022 Jun 2;11(11):1496. doi: 10.3390/plants11111496 (PMC9182668; doi:10.3390/plants11111496)
Supplement: Supplementary file 1 [file plants-11-01496-s001.zip › plants-1726383-supplementary.pdf]

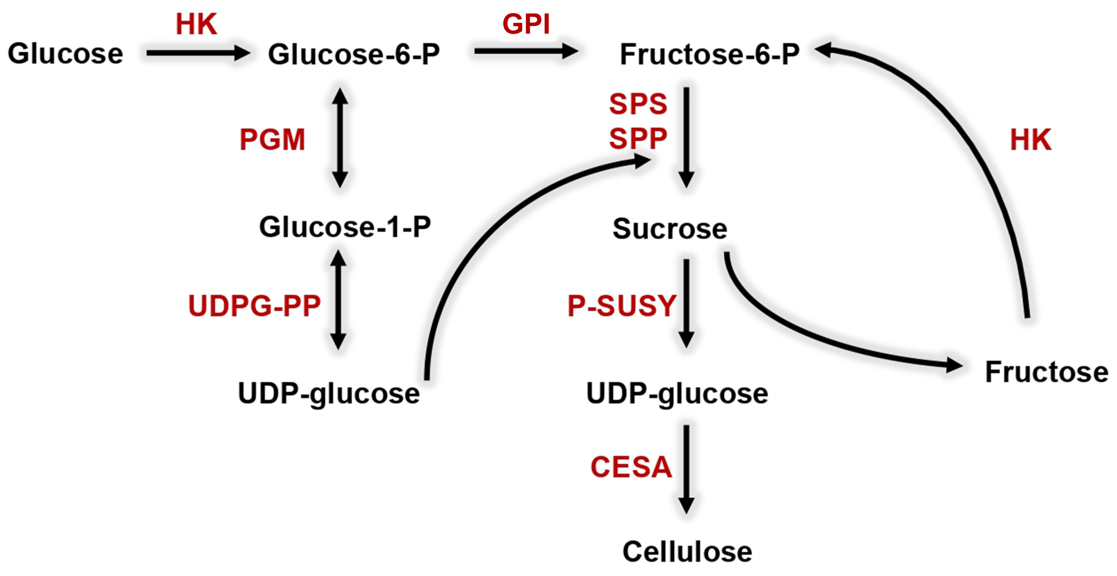

Figure S1. Biosynthetic pathway for cellulose in plants. SUCROSE PHOSPHATE SYNTHASE (SPS), SUCROSE PHOSPHATE PHOSPHATASE (SPP), FRUCTOKINASE (FK), PHOSPHOGLUCOMUTASE (PGM), GLUCOSE-6-PHOSPHATE ISOMERASE (GPI), SUCROSE SYNTHASE (SUS), HEXOKINASE (HXK), UDP-GLUCOSE PYROPHOSPHORYLASE (UGP) AND CELLULOSE SYNTHASE (CESA).

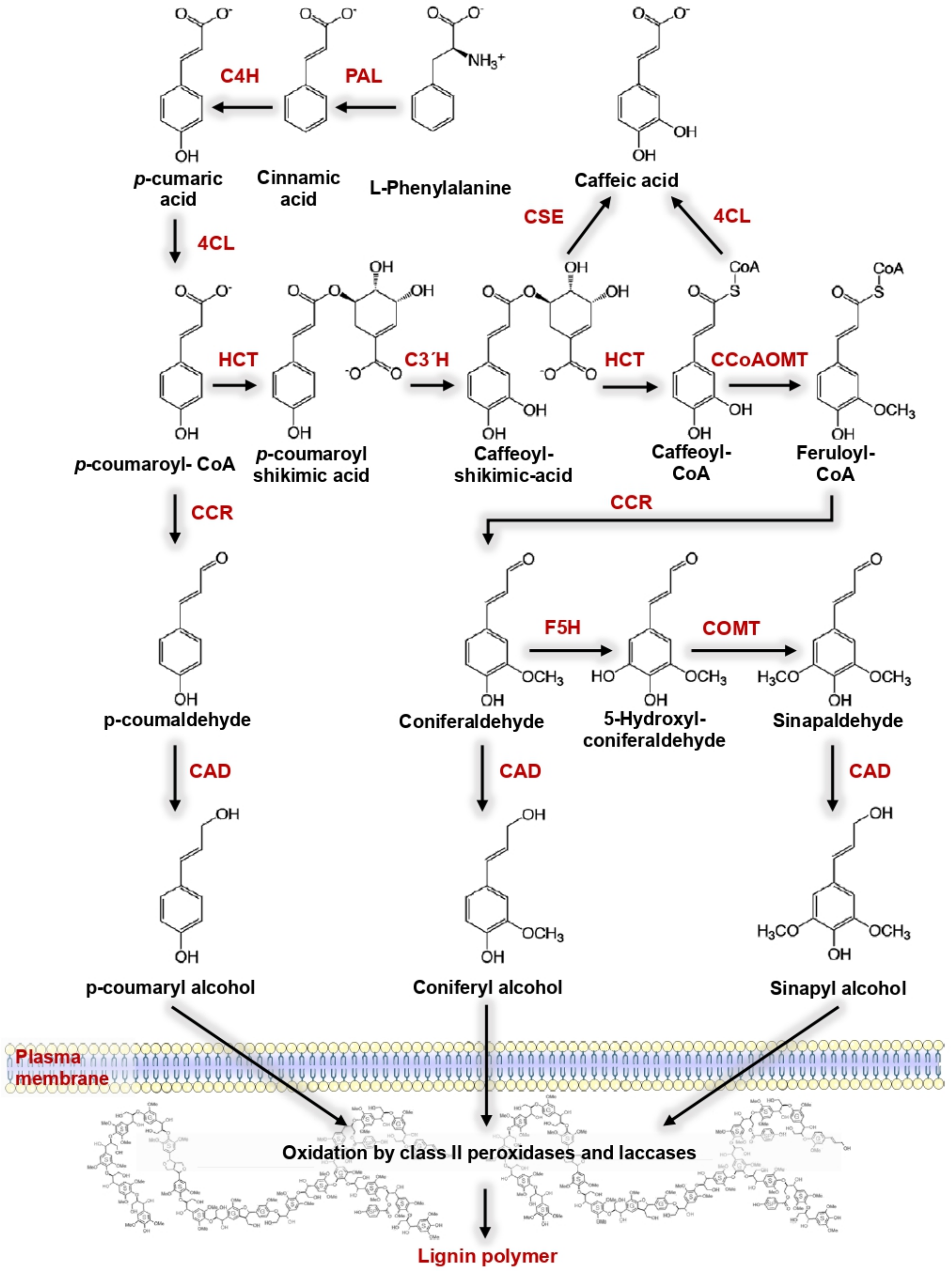

Figure S2. Biosynthetic pathway for lignin in plants. L-PHENYLALANINE AMMONIA-LYASE (PAL), CINNAMIC ACID 4-HYDROXYLASE (C4H), 4-HYDROXYCINNAMATE COA LIGASE (4CL), CAFFEOYL SHIKIMATE ESTERASE (CSE), HYDROXYCINNAMOYL COA: SHIKIMATE HYDROXYCINNAMOYL TRANSFERASE (HCT), COUMARATE 3-HYDROXYLASE (C3H), CAFFEOYL COA 3-O-METHYLTRANSFERASE (CCoAOMT), CINNAMOYL COA REDUCTASE (CRR), FERULIC ACID/CONIFERALDEHYDE 5-HYDROXYLASE (F5H), CAFFEIC ACID 3-O-METHYLTRANSFERASE (COMT) and CINNAMYL ALCOHOL DEHYDROGENASE (CAD).

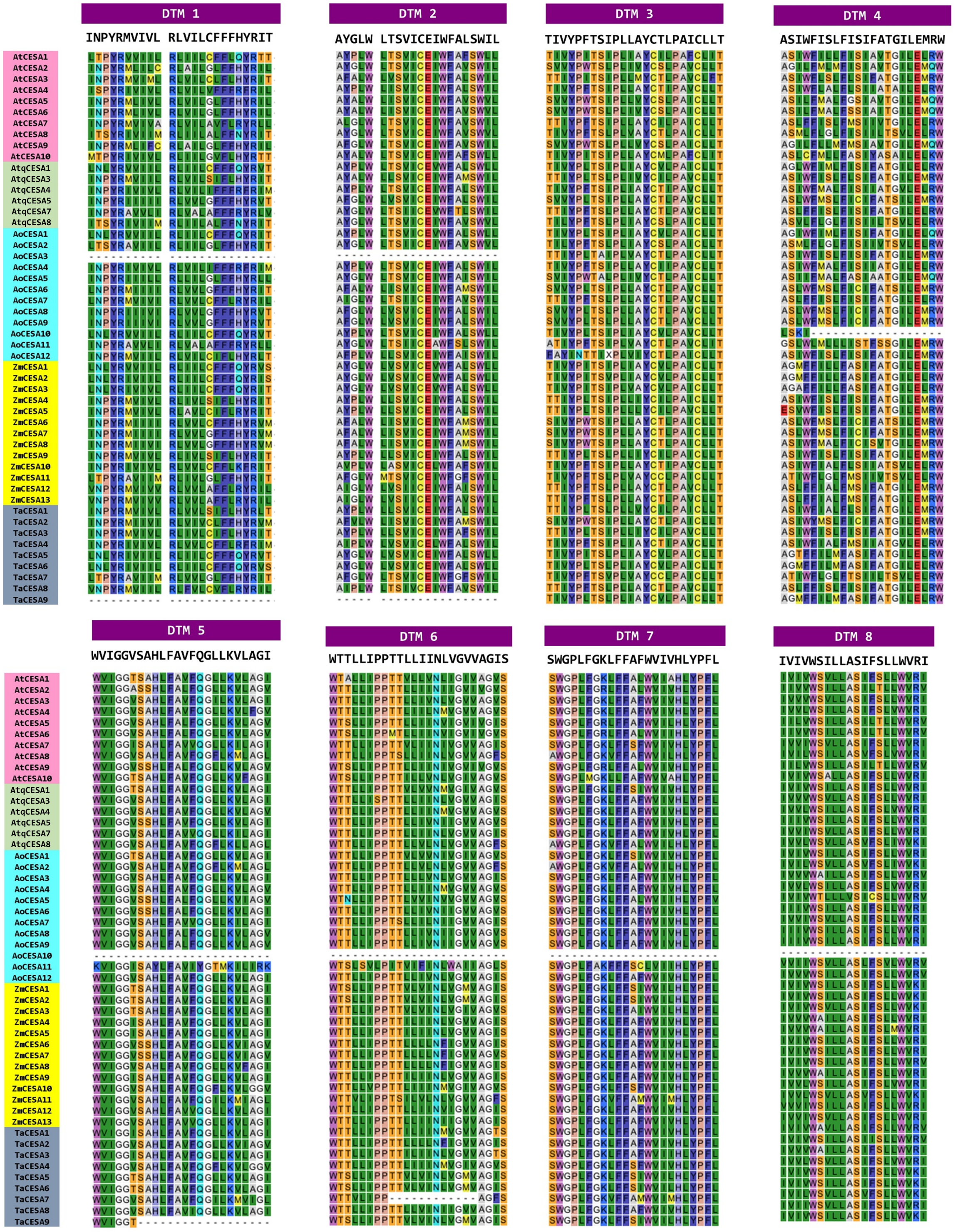

Figure S3. AtqCESA proteins have conserved transmembrane domains. Eight transmembrane domains (TMD) previously characterized in *A. thaliana* (AtCESA) were found to be conserved in *A. tequilana* (AtqCESA) respect to other species analyzed. *A. officinalis* (AoCESA), *Z. mays* (ZmCESA), *T. aestivum* (TaCESA). Consensus residues are indicated for each TMD.

```
                           20                  40                  60                  80                 100
                            |                   |                   |                   |                   |
AtCAD1   -----------------------------------------------------MSSSESVENECMCWAARDPSGLLSPHTITRRSVTTDDVSLTITHCGVCYADVIWSRN  57
AtCAD2   ---------------------------------------------------------MVDQNKAFGWAANDESGVLSPFHFSRRENGENDVTVKILFCGVCHSDLHTIKN  53
AtCAD3   ---------------------------------------------------------MVDQNRAFGWAANDESGVLSPFHFSRRENGENDVTVKILFCGVCHSDLHTIKN  53
AtCAD4   -----------------------------------------------------MGSVEAGEKKALGWAARDPSGVLSPYSYTLRSTGADDVYIKVICCGICHTDIHQIKN  57
AtCAD5   ------------------------------------------------------MGIMEAERKTTGWAARDPSGILSPYTYTLRETGPEDVNIRIICCGICHTDLHQTKN  56
AtCAD6   --------------------------------------------------MERLSGEKEQSVEAFGWAARDSSGHLSPFVFSRRKTGEEEVRVKVLYCGICHSDLHCLKN  60
AtCAD7   -------------------------------------------------------MGKVLEKEAFGLAAKDESGILSPFSFSRRATGEKDVRFKVLFCGICHTDLSMAKN  55
AtCAD8   -------------------------------------------------------MGKVLQKEAFGLAAKDNSGVLSPFSFTRRETGEKDVRFKVLFCGICHSDLHMVKN  55
AtCAD9   ---------------------------------------------------MAKSPETEHPNKVFGWGARDKSGVLSPFHFSRRDNGENDVTVKILFCGVCHTDLHTIKN  59
AtqCAD1  --------------------------------------------------------MDAESGNCLGWAARDTSGALSPYKFSRRSLGNEDISVRITHCGVCYADVIWTRN  54
AtqCAD2  --------------------------------------------MEAKENGKQAAPAAEQRREAVGWAARDSSGILSPFSFSRRNNGDDDVTLKIMYCGICHSDLHSIKN  66
AtqCAD3  ------------------------------------------------------MAMEQEPQKAIGWAAKDSSGVFSPFSFTRRANGDHDVTIRILYCGICHTDLHYAKN  56
AtqCAD4  ---------------------------------------------------------MAELHTAFGWAARDATGILSPFNFSTRAIGDNDVTLKILYCGICHTDLHIIKN  53
AtqCAD5  ------------------------------------------------------MGSVASERTVTGWAAKDASGVLSPYTYSLRPTGAEDVVIKVLYCGVCHTDIHQTRN  56
AtqCAD6  -------------------------------------------MVVAANGTKASSPETEHPRKAFGWAARDSSGVLSPFTFSRRNNGDDDVTLKILYCGICHSDLHTIKN  67
AtqCAD7  ------------------------------------------------------MDTKSGNGDCLGWAARDVSGYLSPYKFSRRVVGSDDVSLKITHCGVCYADVIWTRN  56
ZmCAD1   ---------------------------------------------------MAPVEAEQHRRRALALAAHDASGAVSPIRISRRDTGDDDVAIQILYCGICHSDLHTIKN  59
ZmCAD2   ------------------------------------------------------MAAESENGNCKAWAARDPSGVLSPHSFNRRAVQSGDVSLKIIYCGVCYADVAWTRN  56
ZmCAD3   ------------------------------------------------------MGSLASERKVVGWAARDATGHLSPYSYTLRNTGPEDVVVKVLYCGICHTDIHQAKN  56
ZmCAD4   MSYHCRVLVPVPFHYPPGAGARVGAGAGSPFPSASRALRLPRPRASVEKREQVAAMEEQGGQAALGWAARDDSGVLSPYSFSRRVPKDDDVTIKVLYCGICHTDLHVIKN  110
TaCAD1   -----------------------------------------------------MGSVDASETTVTGWAARDATGHLSPYRYTLRKTGPEDVVLKVKYCGICHTDVHQVKN  57

                 120                 140                 160                 180                 200                 220
                   |                   |                   |                   |                   |                   |
AtCAD1   QHGDSKYPLVPGHEIAGIVTKVGPNVQRFKVGDHVGVGTYVNSCRECEYCNEGQEVNCAK-GVFTFNGIDHDG--SVTKGGYSSHIVVHERYCYKIPVDYPLESAAPLLC  164
AtCAD2   HWGFSRYPIIPGHEIVGIATKVGKNVTKFKEGDRVGVGVIIGSCQSCESCNQDLENYCPK-VVFTYNSRSSDG-TSRNQGGYSDVIVVDHRFVLSIPDGLPSDSGAPLLC  161
AtCAD3   HWGFSRYPIIPGHEIVGIATKVGKNVTKFKEGDRVGVGVIIGSCQSCESCNQDLENYCPK-VVFTYNSRSSDG-TR-NQGGYSDVIVVDHRFVLSIPDGLPSDSGAPLLC  160
AtCAD4   DLGMSNYPMVPGHEVVGEVLEVGSDVSKFTVGDVVGVGVVVGCCGSCKPCSSELEQYCNK-RIWSYNDVYTDG--KPTQGGFADTMIVNQKFVVKIPEGMAVEQAAPLLC  164
AtCAD5   DLGMSNYPMVPGHEVVGEVVEVGSDVSKFTVGDIVGVGCLVGCCGGCSPCERDLEQYCPK-KIWSYNDVYING--QPTQGGFAKATVVHQKFVVKIPEGMAVEQAAPLLC  163
AtCAD6   EWHSSIYPLVPGHEIIGEVSEIGNKVSKFNLGDKVGVGCIVDSCRTCESCREDQENYCTK-AIATYNGVHHDG--TINYGGYSDHIVVDERYAVKIPHTLPLVSAAPLLC  167
AtCAD7   EWGLTTYPLVPGHEIVGVVTEVGAKVKKFNAGDKVGVGYMAGSCRSCDSCNDGDENYCPK-MILTSGAKNFDD--TMTHGGYSDHMVCAEDFIIRIPDNLPLDGAAPLLC  162
AtCAD8   EWGMSTYPLVPGHEIVGVVTEVGAKVTKFKTGEKVGVGCLVSSCGSCDSCTEGMENYCPK-SIQTYGFPYYDN--TITYGGYSDHMVCEEGFVIRIPDNLPLDAAAPLLC  162
AtCAD9   DWGYSYYPVVPGHEIVGIATKVGKNVTKFKEGDRVGVGVISGSCQSCESCDQDLENYCPQ-MSFTYNAIGSDG-TK-NYGGYSENIVVDQRFVLRFPENLPSDSGAPLLC  166
AtqCAD1  KVGDSNYPLVPGHEIVGVVTGFGSNVQRFKVGDHVGVGAYVNSCRDCQYCNEQLEVYCSKGITFTFNAPDSDG--TFTKGGYSSYIVVHNRYCYKIPDGYPLAKAAPLLC  162
AtqCAD2  DWGTTRYPLVAGHEIAGIVTEVGKNVQKFKIGDKVGVGCIVGSCRSCQSCKQDLENYCPN-LIFTYNSIDNDG--TVTYGGYSNMLVVNEHFAVKFPDNLPLDKGAPLLC  173
AtqCAD3  DHGFSLYPIVPGHEIMGEVTRIGSKVQKFKVGDKVGVGCLVRTCGECENCRGDLECYCPK-PVYTYNSVDHDG--SVTYGGYSNQIVVDEHYAIRVPHNLPLDSAAPLLC  163
AtqCAD4  DFGISMYPVVPGHEIVGVVTEAGRNVRKFKRGDKIGVGYMVGSCSACDCCAQGFENYCPK-IVPTSNGIRSDG--TITYGGFADKLVVDEHFAVRLPENLDLDKAAPLLC  160
AtqCAD5  HLGMSNYPMVPGHEVVGEVVEVGSGVGKCRVGDVVGAGCIVGCCRECGPCKANIEQYCSK-KIWSYNDVYTDG--KPTMGGFASAMVVDQKFVVKIPAGLAPEQAAPLLC  163
AtqCAD6  EWQNTSYPVVPGHEIVGVVTEVGSNVNKFKVGDKVGIGCMVESCRSCDSCNQHLENYCPK-IIFTYNSINKDG--TKTYGGYSDMIVVNEHFVVKFPENMPLDKGAPLLC  174
AtqCAD7  KFGDSNYPLVPGHEIVGVVIEIGSNVKHFKVGDHVGVGTYVNSCRDCENCNEYQEIHCSK-ITSTYNSLDLDG--TFTKGGFSNYIIVHDRYCYKIPVGYPLAMAAPLLC  163
ZmCAD1   EWKNANYPVVPGHEIAGLITEVGKNVKRFNVGDKVGVGCMVNTCQSCESCEGGHENYCSK-IIFTYNSHDRDG--TVTYGGYSDMVVVNERFVIRFPDGMPLDRGAPLLC  166
ZmCAD2   KLNDSKYPLVPGHEIAGVVTQVGADVKGFKVGDHVGVGTYVNSCRDCENCNISLDNHCPK-AVYTFNGIDTDG--TVTKGGYSTHIVVHERYCFKIPDGYPLAKAAPLLC  163
ZmCAD3   HLGASKYPMVPGHEVVGEVVEVGPEVAKYGVGDVVGVGVIVGCCRECSPCKANVEQYCNK-KIWSYNDVYTDG--RPTQGGFASTMVVDQKFVVKIPAGLAPEQAAPLLC  163
ZmCAD4   DWRNAMYPVVPGHEIVGVVTGVGGGVTRFKAGDTVGVGYFVGSCRSCDSCGKGDDNYCAG-IVLTSNGVDHAHGGAPTRGGFSDVLVASEHYVVRVPDGLALDRTAPLLC  219
TaCAD1   DLGASKYPMVPGHEVVGEVVEVGPEVSKFRAGDVVGVGVIVGCCRDCRPCKANVEQYCNK-KIWSYNDVYTDG--KPTQGGFASAMVVDQKFVVKIPAGLAPEQAAPLLC  164

                                 240                 260                 280                 300                 320
                                   |                   |                   |                   |                   |
AtCAD1   AGITVYAPMMRHNMN-QPGKSLGVIGLGGLGHMAVKFGKAFGLSVTVFSTSISKKEEALNLLGAENFVISSDHDQMKALEKSLDFLVDTASGDHAFDPYMSLLKIAGTYV  273
AtCAD2   AGITVYSPMKYYGMTKESGKRLGVNGLGGLGHIAVKIGKAFGLRVTVISRSSEKEREAIDRLGADSFLVTTDSQKMKEAVGTMDFIIDTVSAEHALLPLFSLLKVNGKLV  271
AtCAD3   AGITVYSPMKYYGMTKESGKRLGVNGLGGLGHIAVKIGKAFGLRVTVISRSSEKEREAIDRLGADSFLVTTDSQKMKEAVGTMDFIIDTVSAEHALLPLFSLLKVSGKLV  270
AtCAD4   AGVTVYSPLSHFGLM-ASGLKGGILGLGGVGHMGVKIAKAMGHHVTVISSSDKKKEEAIEHLGADDYVVSSDPAEMQRLADSLDYIIDTVPVFHPLDPYLACLKLDGKLI  273
AtCAD5   AGVTVYSPLSHFGLK-QPGLRGGILGLGGVGHMGVKIAKAMGHHVTVISSSNKKREEALQDLGADDYVIGSDQAKMSELADSLDYVIDTVPVHHALEPYLSLLKLDGKLI  272
AtCAD6   AGISMYSPMKYFGLT-GPDKHVGIVGLGGLGHIGVRFAKAFGTKVTVVSSTTGKSKDALDTLGADGFLVSTDEDQMKAAMGTMDGIIDTVSASHSISPLIGLLKSNGKLV  276
AtCAD7   AGVTVYSPMKYHGLD-KPGMHIGVVGLGGLGHVAVKFAKAMGTKVTVISTSERKRDEAVTRLGADAFLVSRDPKQMKDAMGTMDGIIDTVSATHPLLPLLGLLKNKGKLV  271
AtCAD8   AGITVYSPMKYHGLD-KPGMHIGVVGLGGLGHVGVKFAKAMGTKVTVISTSEKKRDEAINRLGADAFLVSRDPKQIKDAMGTMDGIIDTVSATHSLLPLLGLLKHKGKLV  271
AtCAD9   AGITVYSPMKYYGMT-EAGKHLGVAGLGGLGHVAVKIGKAFGLKVTVISSSSTKAEEAINHLGADSFLVTTDPQKMKAAIGTMDYIIDTISAVHALYPLLGLLKVNGKLI  275
AtqCAD1  AGITVYTPMIRHRMN-QAGKSLGVIGLGGLGHMAVKFGKSFGLRVTVFSTSASKKDEALKLLGADEFVVSSDEKEMQSKANTLDFILDTASGDHPFDPYMSLLKVGGVMS  271
AtqCAD2  AGITVYSPMKNFGLN-EPGKHLGVVGLGGLGHVAVKFAKGFGMKVTVISSSPSKEKEAIERLGADSFIVSSNPQQIQAAMGTMDGIINTVSAAHAITPLIFLLKPHGKMI  282
AtqCAD3  AGITVYSPMQYFGLN-RPGLHLGVVGLGGLGHIAVKFGKAFGMKVTVISTSPNKEKEAIERLGTDAFLVSHNEEQMKAAIGSMDGIIDTVSAVHPVAPFIDLLKTHGKLI  272
AtqCAD4  AGVTVYSPMKHFGLN-VPGKHLGVVGLGGLGHVAVKFGKAFGMKVTVISSSPNKEKEAVEHFGADAFLVSRNTEQMKAAIGTMDGIIDTVSASHPVAPLLSLLKMHGKMI  269
AtqCAD5  AGVTVYSPLRHFGLT-DPGLRGGILGLGGVGHMGVKIAKAMGHHVTVISSSDKKRAEAMEHLGADAYLVSSDEAQMATTLDSLDYIIDTVPVFHPLEPYLTLLKVNGKLI  272
AtqCAD6  AGITVYSPMKQFGLN-EPGKHLGVVGLGGLGHVAVKFGKAFGMKVTVISSSPSKEKEAIERLGADAFLISSNPEAMQAAMGTMDGIINTVSAVHSLVPLIFLLKVHGKMI  283
AtqCAD7  AGITVYSPMIQHKMN-QPGKSLGVIGLGGLGHMAVKFGKAFGLKVTVFSTSASKKEEALKLLGADEFVTSSDEKQMQSLKNSLDFIIDTASGDHPFDPYMSLLKTRGVLV  272
ZmCAD1   AGITVYNPMKHHGLN-EAGKHIGVVGLGGLGHVAVKFAKAFGMRVTVISTSPGKREEAMETLGADAFVVSGDANQMKAAKGTMDGIMNTASASMSMYAYLALLKPQGKMI  275
ZmCAD2   AGITVYTPMMRHNMN-QPGKSLGVIGLGGLGHMAVKFGKAFGLKVTVFSTSESKRDEAINLLGADNFVISSDTQQMESLKNSLHFIIDTASGDHPFDPYFSLLKVGGVMT  272
ZmCAD3   AGVTVYSPLKHFGLT-TPGLRGGILGLGGVGHMGVKVAKAMGHHVTVISSSSKKRAEAMDHLGADAYLVSSDAAAMGPAADSLDYIIDTVPVHHPLEPYLALLKLDGKLV  272
ZmCAD4   AGVTVYSPMMRHGLN-EPGKHLGVVGLGGLGHVAVKFGKAFGMKVTVISTSASKRQEAIENLGADEFLISRDEDQMKAATGTMDGIIDTVSAWHPITPLLALLKPLGQMV  328
TaCAD1   AGVTVYSPLKHFGLM-TPGLRGGILGLGGVGHMGVKVAKSMGHHVTVISSSNKKRAEAMDDLGADAYLVSSDTDQMAAAADSLDYIIDTVPAKHPLEPYLALLKMDGKLV  273
```

```
                    340                 360                 380                 400                 420
                      |                   |                   |                   |                   |
AtCAD1   LVGFPS-EIKISPANLNLGMRMLAGSVTGGTKITQQMLDFCAAHKIYPNIEVIPIQKINEALERVVKKDIKYRFVIDIKNSLK----------------------      355
AtCAD2   ALGLPEKPLDLPIFSLVLGRKMVGGSQIGGMKETQEMLEFCAKHKIVSDIELIKMSDINSAMDRLAKSDVRYRFVIDVANSLLPESSAEILTEQVDHGVSITSRF      376
AtCAD3   ALGLLEKPLDLPIFPLVLGRKMVGGSQIGGMKETQEMLEFCAKHKIVSDIELIKMSDINSAMDRLVKSDVRYRFVIDVANSLLPESSAEILTEHVDHGVSITSRF      375
AtCAD4   LMGVINTPLQFVTPLVILGRKVISGSFIGSIKETEEVLAFCKEKGLTSTIETVKIDELNIAFERLRKNDVRYRFVVDVAGSNLVEEAATTTN-------------      365
AtCAD5   LMGVINNPLQFLTPLLMLGRKVITGSFIGSMKETEEMLEFCKEKGLSSIIEVVKMDYVNTAFERLEKNDVRYRFVVDVEGSNLDA--------------------      357
AtCAD6   LLGATEKPFDISAFSLILGRKSIAGSGIGGMQETQEMIDFAAEHGIKAEIEIISMDYVNTAMDRLAKGDVRYRFVIDISNTLAATRS------------------      363
AtCAD7   MVGAPAEPLELPVFPLIFGRKMVVGSMVGGIKETQEMVDLAGKHNITADIELISADYVNTAMERLAKADVKYRFVIDVANTMKPTP-------------------      357
AtCAD8   MVGAPEKPLELPVMPLIFERKMVMGSMIGGIKETQEMIDMAGKHNITADIELISADYVNTAMERLEKADVRYRFVIDVANTLKPNPNL-----------------      359
AtCAD9   ALGLPEKPLELPMFPLVLGRKMVGGSDVGGMKETQEMLDFCAKHNITADIELIKMDEINTAMERLAKSDVRYRFVIDVANSLSPP--------------------      360
AtqCAD1  LVGFPS-EIKMHPGSLIYGARTISGSMTGGTKDTQEMLDFCAANKIYPDIEVIPIQYINEALERLIKKDVKYRFVIDIENSLK----------------------      353
AtqCAD2  LVGLPDKPFELPAYSLILGGKILAGSVMGGLKDTQEMIDFAGKHNITADIELICMDDVNKAMERLAKGDVRYRFVIDVASS------------------------      363
AtqCAD3  VVGGIAEPLSVPTIPLLLGRKLVAGSAIGGIKETEEMIEFAAKHNIMADIEVIPIDYISTAMERLAKGDVRYRFVIDVANTL-----------------------      354
AtqCAD4  NVGLPTKPMEVPAFSLIQGGRTLAGSYVGGMRDTQEMIDFAGKHNITAEVEVVSMDYVNTAFERLAKGDVRYRFVIDVANSLTPA--------------------      354
AtqCAD5  MMGVINQPMQFITPMVMLGRKTITGSFIGSMDETQEVLEFCEKKGLTSMIEMVKMDQVNEALERLERNDVRYRFVVDVAGSNLD---------------------      356
AtqCAD6  MVGAPEKPFELPVFPLIMAGKVLAGSCIGGMKDTQEMIDFAGKHNVTADIELINMDYVNKAMERLAKADVRYRFVIDVANSLIAADQ------------------      370
AtqCAD7  LVGAPS-EVKVEPRSLIHGARTISGSATGGTKDTKEMLDFCAAKKIYPEIEVIPIQYINEALERMIKRDVKYRFVIDIENSLK----------------------      354
ZmCAD1   LLGLPEKPLQISAFSLVTGGKTLAGSCMGSIRDTQEMMDFAAKHGLAADIELIGTEEVNEAMERLAKGEVRYRFVIDIGNTLNAASLGSSPVPAL----------      370
ZmCAD2   LVGFPS-EIKMHPASLILGARTLSGSVVGGIKDIQEMVNFCAANNIYPEIEIIKIDYINEALARLVNRDVKYRFVIDIENSFK----------------------      354
ZmCAD3   LLGVIGEPLSFVSPMVMLGRKAITGSFIGSIDETAEVLQFCVDKGLTSQIEVVKMGYVNEALERLERNDVRYRFVVDVAGSNVEAEAAAADAASN----------      367
ZmCAD4   VVGAPSKPLELPAYAIVPGGKGVAGNNVGSVRDCQAMLEFAGKHGIGAEVEVIKMDYVNTAMERLEKNDVRYRFVIDVAGSLGSAA-------------------      414
TaCAD1   LMGVIAEPLSFVSPMVMLGRKTITGSFIGSMDETEEVLQFCVDKGLTSQIEVVKMDYVNQAFERLERNDVRYRFVVDVGGSNIEDAA------------------      360
```

Figure S4. Protein alignment for AtqCAD. Residues that compose the substrate-binding pocket of CAD proteins are highlight in color.

Table S1. Expression levels of genes involved in cellulose biosynthesis in *Agave tequilana* in several organs and tissues sampled. Values shown are transcripts per million (TPM).

Gene name	Root	Pine	Leaves	Shoot apical meristem	Anther	Ovary	Tepal	Pistil
SPS1	0	45.2085807	0	33.3521462	42.7731031	33.9497662	53.4816066	65.6248299
SPS2	19.9594525	62.7254275	468.275016	29.4269683	185.443037	290.997996	356.030153	426.843449
SPP1	230.695629	100.550917	151.241681	122.924041	76.4881824	146.36058	113.69358	142.560071
SPP2	13.1874954	17.3677454	7.38183532	22.1081965	18.0150436	14.8979562	26.4107889	22.9780923
FK1	23.5236405	8.74042843	13.0076393	2.84777113	3.63613798	0.57058431	30.6841143	18.8036762
FK2	4.72611322	1.39332712	1.39367564	11.215472	4.4562891	0	0.48177287	4.70091905
FK3	126.885091	26.7354281	456.661052	68.3465071	50.097901	11.4116861	32.2787825	20.6840438
FK4	39.2060674	34.9617137	97.5572949	34.647882	48.0778243	49.0702503	69.3752936	78.5053481
FK5	81.0781475	53.275482	1.99760175	21.7712103	13.3890681	6.52748446	2.69311036	9.87663091
GPI1	146.131706	106.42757	83.6205385	140.015414	49.2898703	79.8818028	76.1201139	105.770679
PGM1	4.27702554	10.8175655	71.5373707	19.3885751	23.5056119	25.1456503	33.5843869	38.035136
PGM2	253.770182	251.929996	757.230432	234.466489	331.292571	297.845007	616.187504	688.214548
SUS1	17.3504669	9.56305699	1.20320664	35.9009013	7.13895089	42.2403561	9.51501423	25.0417958
SUS2	328.354379	235.17408	484.441653	30.1294185	1185.68399	2065.28695	2812.84055	4266.17805
SUS3	90.5303739	179.949997	15.3304321	179.409581	5.65621463	18.8292821	5.2995016	8.93174619
HXK1	4.27702554	5.85094562	2.81987038	6.9960244	109.544717	5.34637494	8.4695671	4.05689314
HXK2	7.16401778	12.1337712	0.95234502	11.732817	0.92115495	4.10250116	6.8604457	8.07617892
UGP1	335.033667	344.475709	957.919725	264.368086	270.286256	516.949381	660.028835	841.464509
UGP2	7.49192307	11.5219412	3.25190983	18.1450484	52.1179776	7.29777327	7.22659309	24.0310982
UGP3	0	0	6.77326362	2.8240397	0	0	0	1.11881873
CESA1	550.039741	265.667893	369.630654	286.699358	243.811132	190.854744	244.114315	732.788663
CESA3	424.159751	230.09949	232.990048	339.065123	116.126126	53.4922786	130.179848	442.041521
CESA4	223.118166	173.26614	229.491922	164.696097	1030.23909	87.8699831	187.89142	286.756062
CESA5	125.26695	2.00001568	65.7303889	1.42863185	2.02007665	0	0	1.88036762
CESA7	88.2920639	3.3573528	69.9857452	0	0	0	0	0.75684797
CESA8	201.597599	169.404927	111.865698	91.1998703	59.9558751	104.610927	76.1586557	125.664968

Table S2. Expression levels of genes involved in lignin biosynthesis in *Agave tequilana* in several organs and tissues sampled. Values shown are transcripts per million (TPM).

Gene name	Root	Pine	Leave	Shoot apical meristem	Anther	Ovary	Tepal	Pistil
PAL1	203.728983	15.0746683	28.2358685	9.00844933	4.18155867	9.60863971	17.7003353	30.3444324
PAL2	67.9761926	12.8175812	1.8535886	7.22859238	204.302472	78.6493407	185.72826	306.546931
C4H	353.595958	18.1698083	54.4787808	7.07196497	35.2260967	27.8102791	67.2362221	133.181737
4CL	58.4526824	34.9617137	5.57470257	18.0358838	90.9034494	13.6940233	66.9664293	157.010696
CSE	44.6236331	18.0721211	10.9821641	3.9156853	12.2901464	12.9237345	25.697765	11.1834864
HCT1	88.5486854	25.7688396	16.3663976	0.8353462	2.25036539	12.3873853	35.8439017	6.13469935
HCT2	3.11510027	0	0	0	0	0	0	0
C3H	164.665483	24.6788567	19.0469004	14.7134842	23.4328892	7.98818028	30.8334638	29.1456981
CcoAOMT	368.216257	15.851024	40.8579242	6.78718785	0	6.1623105	7.72281915	14.7843904
CCR1	9.3453008	0	0.24621603	0	0	0	0	0
CCR2	110.489826	56.5557134	27.4089543	21.3582835	11.3124293	7.41759597	31.7970096	13.6326652
F5H	112.628339	20.5657139	106.383907	0.94925704	37.3835385	2.85292153	25.4906027	10.6804881
COMT1	409.468168	152.278829	227.917069	52.270839	42.0781967	54.6905057	76.0671188	98.31032
COMT2	0	0.51414285	449.228115	34.647882	122.820661	82.16414	178.255963	314.961576
CAD1	45.2794437	32.0362409	33.5550639	31.235303	48.2232699	20.5125058	60.4817664	39.0035253
CAD2	0.71283759	2.57071424	6.96837821	36.0717676	0.80803066	18.2586978	1.44531862	8.93174619
CAD3	57.7398448	11.7070327	4.64558547	17.5517627	30.0587406	26.5207585	264.878725	75.1676955
CAD4	3.56418795	3.74810137	42.8694628	5.22091373	0.80803066	1.71175292	6.8267216	2.35045952
CAD5	137.313905	74.9825931	58.1906036	27.0016166	0	15.9478313	12.9596903	5.83854145
CAD6	0	1.6606814	0	9.97669152	4.2825625	48.2257855	3.46876468	2.86756062
CAD7	34.9290419	0.51414285	18.5823419	0.47462852	12.9284906	5.13525875	24.0886436	21.6242276

Table S3. Percentages of identity/similarity between amino acid sequences of orthologous *CESA* genes of *A. tequilana* (AtqCESA), *A. officinalis* (AoCESA), and *A. thaliana* (AtCESA)

	**AtqCESA1**	**AtqCESA3**	**AtqCESA4**	**AtqCESA5**	**AtqCESA7**	**AtqCESA8**
**AoCESA1**	93/96	66/74	61/71	65/75	61/72	56/67
**AoCESA2**	58/67	58/68	62/72	58/68	56/67	85/89
**AoCESA3**	50/55	58/60	49/54	48/54	48/53	47/54
**AoCESA4**	60/71	61/71	91/94	64/74	62/73	61/72
**AoCESA5**	60/71	58/68	60/71	70/80	59/72	53/66
**AoCESA6**	67/75	63/72	63/73	90/93	68/78	56/67
**AoCESA7**	63/72	65/72	63/72	68/77	77/85	57/67
**AoCESA8**	67/76	62/71	64/73	92/95	68/78	56/67
**AoCESA9**	65/74	61/70	63/72	90/93	67/77	55/66
**AoCESA10**	78/80	52/59	47/56	53/61	48/57	45/53
**AoCESA11**	47/60	48/60	45/59	46/58	53/63	44/57
**AoCESA12**	69/77	81/86	61/70	63/74	61/71	57/68
**AtCESA1**	82/89	64/74	60/71	64/74	60/72	56/66
**AtCESA2**	61/73	60/70	61/73	71/82	61/74	54/67
**AtCESA3**	69/78	77/84	61/69	63/72	61/72	57/68
**AtCESA4**	58/70	61/71	77/84	60/71	58/70	60/71
**AtCESA5**	62/72	60/70	62/73	70/81	60/75	55/68
**AtCESA6**	61/72	59/69	62/73	71/82	60/74	54/67
**AtCESA7**	62/71	63/72	64/73	66/76	73/83	56/67
**AtCESA8**	57/67	57/67	62/71	57/67	56/67	84/90
**AtCESA9**	61/72	59/69	60/71	71/80	60/73	55/66
**AtCESA10**	76/84	63/73	57/69	61/72	57/69	54/66

Table S4. Percentages of identity/similarity between amino acid sequences of orthologous CAD genes of *A. tequilana* (AtqCAD), *O. sativa* (OsCAD), and *A. thaliana* (AtCAD)

	AtqCAD1	AtqCAD2	AtqCAD3	AtqCAD4	AtqCAD5	AtqCAD6	AtqCAD7
**OsCAD1**	72/84	51/64	52/66	49/65	45/63	49/64	72/82
**OsCAD2**	41/60	45/62	48/65	48/65	80/88	45/62	42/59
**OsCAD3**	51/64	65/77	58/73	61/75	45/62	67/78	50/65
**OsCAD4**	69/79	47/62	48/63	45/62	41/58	47/62	70/80
**OsCAD5**	46/59	62/72	54/67	60/71	43/57	65/75	46/60
**OsCAD6**	49/64	54/69	54/70	56/69	48/64	56/69	50/64
**OsCAD7**	44/59	61/75	54/69	54/69	42/59	62/76	42/60
**OsCAD8A**	46/61	55/70	56/71	61/73	48/62	59/71	47/64
**OsCAD8B**	40/52	50/61	47/59	53/63	40/52	51/63	39/52
**OsCAD8C**	40/52	49/60	47/59	52/64	40/52	50/62	39/52
**OsCAD8D**	45/60	55/70	55/70	61/73	48/61	58/71	45/62
**OsCAD9**	45/61	54/72	56/71	59/75	46/66	58/73	45/62
**AtCAD1**	71/81	48/64	50/66	45/63	41/59	46/63	71/81
**AtCAD2**	47/60	56/70	55/69	53/70	45/62	57/70	45/59
**AtCAD3**	46/60	55/69	55/69	52/69	45/62	56/70	45/58
**AtCAD4**	43/61	45/63	48/66	46/63	73/82	46/63	45/59
**AtCAD5**	44/62	45/63	51/67	48/65	75/83	47/64	46/62
**AtCAD6**	46/61	59/75	60/76	58/76	47/65	60/76	48/64
**AtCAD7**	47/62	59/72	60/74	63/77	47/62	62/74	48/64
**AtCAD8**	46/60	60/73	61/74	60/75	48/63	64/74	47/61
**AtCAD9**	49/64	62/77	60/75	60/74	48/65	65/77	47/62

Table S5. cDNA libraries used in this study, previously reported by Gross et al., 2013 and deposited in Genbank (SRP019885)

cDNA library name	Read length (pb)	Tissue or organ sequenced
SRX253025	500	Juvenil leaf
SRX253024	250	Juvenil leaf
SRX253023	500	Roots
SRX253022	250	Roots
SRX253021	500	Stem apical meristem
SRX253020	250	Stem apical meristem
SRX253019	500	Adult leaf
SRX253018	250	Adult leaf

Table S6. Gene-specific oligonucleotides used for qPCR analysis

Gene name	Sequence (5'-3')
AtqSUS2	F: GAATGCTGCAACGCCATCAA<br>R: CTTAGAGCTGCCATCTGCGA
AtqCESA1	F: GTGCCAGATCTGTGGTGACA<br>R: CTGGTCAGACGTGGGATACG
AtqCESA7	F: GGCCCCGAGGATGTTGATAG<br>R: GGGACGACGAAGAGAAGTCG
AtqCCR2	F: GCCCATTTGGTGGTCTACGA<br>R: GTACGCCCAGGTCTCTTAGC
AtqCD5	F: TGTGTTCCATCCCTTGGAGC<br>R: CGAGATTGCTTCCAGCAACG
AtqGAPDH	F: ACACTGTCCACGGTTCATGG<br>R: TCCACAACGTACTCAGCACC

F= Forward; R= Reverse
